# COX-2 Expression in Hepatocytes Improves Mitochondrial Function after Hepatic Ischemia-Reperfusion Injury

**DOI:** 10.3390/antiox11091724

**Published:** 2022-08-30

**Authors:** Marina Fuertes-Agudo, María Luque-Tévar, Carme Cucarella, Rocío Brea, Lisardo Boscá, Rubén Quintana-Cabrera, Paloma Martín-Sanz, Marta Casado

**Affiliations:** 1 Instituto de Biomedicina de Valencia (IBV), CSIC, Jaume Roig 11, 46010 Valencia, Spain; 2 Centro de Investigación Biomédica en Red de Enfermedades Hepáticas y Digestivas (CIBERehd), Monforte de Lemos 3-5, 28029 Madrid, Spain; 3 Instituto de Investigaciones Biomedicas Alberto Sols (IIBM), CSIC-UAM, Arturo Duperier 4, 28029 Madrid, Spain; 4 Centro de Investigación Biomédica en Red de Enfermedades Cardiovasculares (CIBERcv), Monforte de Lemos 3-5, 28029 Madrid, Spain; 5 Instituto Cajal (IC), CSIC, Avda. Doctor Arce, 237, 28002 Madrid, Spain

**Keywords:** COX-2, prostaglandins, liver, ischemia-reperfusion, high-resolution respirometry, mitochondrial dynamics

## Abstract

Cyclooxygenase 2 (COX-2) is a key enzyme in prostanoid biosynthesis. The constitutive hepatocyte expression of COX-2 has a protective role in hepatic ischemia-reperfusion (I/R) injury (IRI), decreasing necrosis, reducing reactive oxygen species (ROS) levels, and increasing autophagy and antioxidant and anti-inflammatory response. The physiopathology of IRI directly impacts mitochondrial activity, causing ATP depletion and being the main source of ROS. Using genetically modified mice expressing human COX-2 (*h-COX-2 Tg*) specifically in hepatocytes, and performing I/R surgery on the liver, we demonstrate that COX-2 expression has a beneficial effect at the mitochondrial level. Mitochondria derived from *h-COX-2 Tg* mice livers have an increased respiratory rate associated with complex I electron-feeding pathways compared to Wild-type (*Wt*) littermates, without affecting complex I expression or assembly. Furthermore, *Wt*-derived mitochondria show a loss of mitochondrial membrane potential (ΔΨm) that correlates to increased proteolysis of fusion-related OPA1 through OMA1 protease activity. All these effects are not observed in *h-COX-2 Tg* mitochondria, which behave similarly to the Sham condition. These results suggest that COX-2 attenuates IRI at a mitochondrial level, preserving the proteolytic processing of OPA1, in addition to the maintenance of mitochondrial respiration.

## 1. Introduction

Orthotopic liver transplantation (OLT) remains the standard treatment for patients with end-stage liver disease and irreversible liver failure. Many patients with end-stage liver disease have benefited from whole-organ liver transplantation because OLT has the added benefit of curing the underlying liver disease [1]. The growing success of OLT is being met with an increasing shortage of available organs. Therefore, the wider use of marginal organs in recent years has been accompanied by a resurgence of interest in strategies to optimize the condition of available organs during the preservation period. Innovative strategies, such as living donation or the splitting of cadaveric or steatotic grafts, are necessary to expand the donor pool. However, these alternatives are more susceptible to ischemia-reperfusion (I/R) injury (IRI), which makes it necessary to find strategies to improve outcomes for OLT.

Hepatic IRI is a major cause of morbidity and mortality in liver resection and liver transplantation. The pathophysiology of IRI includes several mechanisms that contribute to various degrees in the overall injury [2]. Prolonged organ ischemia reduces tissue oxygenation, resulting in tissue ATP depletion with a transition to activation of anaerobic metabolic pathways, which cannot maintain cellular function and ultimately leads to cell death. Restoration of blood flow is necessary to restore cellular function, but paradoxically reperfusion initiates a cascade of pathways that cause further cell injury. After oxygen re-entry, uncoupled dysfunctional mitochondria produce large amounts of oxygen-free radicals, intense oxidative stress and mitochondrial permeability transition leading to cell death. Activation of Kupffer cells also occurs, leading to abundant production of reactive oxygen species (ROS), pro-inflammatory cytokines, and cyclooxygenase 2 (COX-2) derived prostanoids [3,4].

Mitochondria are known to play an important role in IRI damage by increasing oxidative stress, uncoupling metabolic state and inducing apoptosis. During the ischemic phase, intracellular hypoxia stimulates ROS production via complex III of the mitochondrial electron transport chain (ETC) [5]. During the reperfusion phase, oxygen is rapidly reintroduced into cells that have switched to anaerobic respiration. Oxidative phosphorylation (OXPHOS) is disrupted, resulting in the accumulation of reduced electron-carrying molecules in the mitochondria, and ROS production is triggered as reduced electron-carrying molecules donate their electrons to oxygen [6].

In the liver and various tissues, it has been shown that a short period of ischemia protects efficiently against subsequent IRI. This phenomenon, known as ischemic preconditioning (IP), indicates that a brief ischemic insult triggers a protective biological reaction in the liver which is associated with the inhibition of pro-apoptotic pathways [7]. It has been shown that mitochondrial bioenergetics is involved in the protective effect [8]. Mitochondrial-specific targets include respiratory chain enzyme complexes, OXPHOS, and ion channels localized on the inner mitochondrial membrane [9].

Mitochondria are dynamic organelles that contain an outer membrane (OMM), the main platform for mitochondrial signaling, and a protein-rich inner membrane (IMM) mainly involved in mitochondrial energy conversion [10]. The IMM includes two main subcompartments: the inner boundary membrane (IBM) and the mitochondrial cristae, membrane invaginations that harbor components required for OXPHOS and ATP production [11]. Mitochondrial cristae are dynamic structures that adapt their architecture in response to different stimuli and stress. Dynamin-related GTPase optic atrophy type 1 (OPA1) is a key regulator of cristae architecture, which is important for the formation and maintenance of cristae junctions [12]. The length, shape, size and number of mitochondria are controlled by fusion and fission mechanisms, and cells must balance both events to preserve mitochondrial integrity [13]. In mammalian cells, mitochondrial fusion is regulated by two mitofusins, MFN1 and MFN2, which cooperate with OPA1 in the fusion process [14]. In the OMM, mitochondrial fission is orchestrated by the recruitment and mitochondrial assembly of cytosolic dynamin-related protein 1 (DRP1, also known as DNM1L), and dynamin-2 (DYN2), with the assistance of MFF and MIEF1/2 (MiD51/49), which are anchored in the OMM and serve to mediate recruitment of DRP1 to the mitochondrial surface [15]. Furthermore, in mammals, contrary to yeast, FIS1 would act by binding to and blocking the GTPase activity of MFNs and OPA1 (but not DRP1), promoting unopposed fragmentation [16]. Due to proteolytic processing, OPA1 can be found as multiple isoforms, so the long (L-OPA1) isoform mediates IMM fusion and preserves cristae structure, while the short isoform (S-OPA1) generated by proteolysis of the long isoform is required to form oligomers with L-OPA1 for cristae maintenance, but in excess may facilitate mitochondrial fission [10]. Several proteases have been implicated in the constitutive and stress-induced processing of OPA1. These include the i-AAA protease YME1L that constitutively cleaves L-OPA1 isoforms at the S2 proteolytic site [17,18,19], while the metallopeptidase OMA1 processes OPA1 at the S1 site.

Mitochondrial respiration is a key element of cell physiology. Respirometry reflects the function of mitochondria as structurally intact organelles. Measurement of respiratory flux in different metabolic states is required for the evaluation of the effect on oxidative phosphorylation of changes in metabolite levels, membrane permeability, or activity of individual enzymes. The fatty acid oxidation (FAO) pathway control state (F-pathway) feeds electrons into the F-junction through fatty acyl CoA dehydrogenase (reduced form FADH_2_), to the electron transfer flavoprotein complex (CETF), and further through the Q-junction to complex III when fatty acids and low malate concentration (0.1 mM) are supplied in mitochondrial preparations. The NADH electron transfer-pathway state (N-pathway) is obtained by the addition of NADH-linked substrates (CI-linked) [20].

The mitochondrial ETC is the main generator of mitochondrial membrane potential (ΔΨm), through the pumping of protons from the inner matrix to the intermembrane space as an effect of the passage of electrons from one complex to another. The ΔΨm is then used by the ATP synthase, which uses this electronic and chemical potential as fuel to rotate and generate ATP from ADP and inorganic phosphate. A consequence of a decrease in the activity of one of the ETC complexes is the loss of this ΔΨm and, consequently, a decrease in ATP synthesis [21].

Cyclooxygenase (COX) is a key regulatory step in the biosynthesis of prostanoids. Prostaglandins (PGs) are active lipid components involved in several homeostatic processes, such as platelet aggregation, vasodilatation and vasoconstriction among others, as well as playing an important role in the onset of inflammation. COX-2 is expressed and induced by different stimuli in various tissues and cell types; however, in the liver, COX-2 expression is restricted to those situations where proliferation or dedifferentiation occur [22,23]. The pathophysiological role of COX-2 expression has been analyzed in studies in which its activity has been reduced by chemical inhibitors or by using knockout mouse models. However, very few studies have investigated the effects of constitutive hepatic expression of COX-2. We have generated the first transgenic (Tg) mouse model carrying the human (h)COX-2 gene (*PTGS2*) under the control of the human Apo E promoter and its endogenous hepatic control region (*h-COX-2 Tg*) [24].

Controversial results related to COX-2 in mitochondrial function and IRI have been published. Non-steroidal anti-inflammatory drugs and selective COX-2 inhibitors have been reported to have significant effects on cellular energy metabolism [25]. For example, flurbiprofen and meloxican protect mice from hepatic IRI by inhibiting the mitochondrial permeability transition and attenuating TNF-α release [26,27]. In contrast, our group has demonstrated that constitutive expression of human COX-2 in transgenic (*h-COX-2 Tg*) mice leads to a lower degree of necrosis and inflammation than in Wild-type (*Wt*) mice after I/R, in part by reducing hepatic recruitment and neutrophil infiltration, with a concomitant decrease in serum levels of pro-inflammatory cytokines. Furthermore, *h-COX-2 Tg* mice showed a significant attenuation of IRI-induced increased oxidative stress and hepatic apoptosis, increased autophagic flux and decreased endoplasmic reticulum stress compared to that observed in *Wt* mice. Moreover, IP of *Wt* mice resembles the beneficial effects observed in *h-COX-2 Tg* mice against IRI due to the preconditioning-derived increase in the endogenous COX-2 expression [28].

Considering the protective effect of hepatic COX-2 induction and the subsequent increase in COX-2-derived PGs against IRI partly due to a decrease in oxidative stress, and the role of mitochondria in IRI, we aimed to elucidate whether constitutive COX-2 expression in the hepatocyte alters mitochondrial function as a further mechanism of the observed protective effect. Our results demonstrate that COX-2 has a positive impact on the maintenance of respiratory coupling, paralleled by the preservation of OPA1 configuration and lower OMA1 cleavage.

## 2. Materials and Methods

### 2.1. Experimental Design

Male *h-COX-2 Tg* mice aged 8 to 10 weeks and their wild-type (*Wt*) siblings (B6D2RccHsd-Tg (APOE-*PTGS2*)4/Upme) were used. Only male mice were used in procedures to avoid hormonal modulation of endogenous prostaglandin levels. Mice were randomly divided into two groups: the sham surgery group and the hepatic ischemia-reperfusion (I/R) group. Thirty minutes before surgery, 100 μL of an analgesic solution (Buprenorphine (Bupaq, CN578816.6, Richter Pharma) 0.05 mg/kg) was injected subcutaneously. The animals were anesthetized with 1.2–2% isoflurane (Isoflutek, CN586259.0, Karizoo), and a model protocol of segmental (70%) warm hepatic I/R for 90 min was performed by clamping the hepatic triad [7]. Reperfusion was initiated by the removal of the clamp for 4 h; 300 μL of warm saline were injected subcutaneously before awakening. The animals were sacrificed by cervical dislocation, opened at the abdominal level and the livers were removed. All animal experimentation was controlled following the recommendations of the Federation of European Laboratory Animal Science Associations on health monitoring, European Community Law (2010/63/UE), and Spanish law (R.D. 53/2013) with approval of the Ethics Committee of the Spanish National Research Council, Spain.

### 2.2. Mitochondria Isolation

Liver mitochondria were isolated as previously described [29]. The left lobe was kept in cold isolation buffer (IB, 0.2 M sucrose, 10 mM Tris-MOPS, 1 mM EGTA-Tris, pH 7.4). After being weighed (mean weight 300–500 mg), the tissue was washed three times with IB, cut into small pieces (~0.5 cm^2^) and homogenized in a glass potter with 5 mL of cold IB at 100× *g*. The homogenate was transferred to a 50 mL centrifuge tube and centrifuged at 600× *g* for 10 min at 4 °C. The pellet was discarded and the supernatant was transferred to a centrifuge tube and centrifuged at 7000× *g* for 10 min at 4 °C. The supernatant was then discarded; the pellet was resuspended in 5 mL of cold IB and centrifuged again at 7000× *g* for 10 min at 4 °C. The supernatant was discarded and the mitochondrial pellet was suspended in 1 mL of IB. The mitochondrial protein concentration was determined using the DC Protein Assay (BioRad, Hercules, CA, USA).

### 2.3. High Resolution Respirometry and ROS Production Evaluation

An Oroboros Oyxgraph-2k FluoRespirometry (O2k, Oroboros Instruments, Innsbruck, Austria) was used for high-resolution respirometry assays. Chambers A and B were loaded with 2 mL of MiR05 medium (0.5 mM EGTA, 3 mM Cl_2_Mg, 60 mM lactobionic acid, 20 mM taurine, 10 mM KH_2_PO_4_, 20 mM HEPES, 100 mM D-sucrose, and 1 g/L fatty-acid free BSA, Oroboros Instruments) and calibrated at 30 °C; 50 μg of mitochondria were loaded into each chamber. All experiments were performed comparing *Wt*- and *h-COX-2 Tg*-derived mitochondria. A substrate-uncoupler-inhibitor-titration protocol termed SUIT-RP2 [30] was performed. The substrates used were: 5 mM ADP, 0.1 mM malate, and 0.2 mM octanoyl-carnitine, to record the electron transfer flavoprotein complex (CETF) from fatty acid β oxidation to coQ (F pathway); 2 mM malate, 5 mM pyruvate, 10 mM glutamate to record OXPHOS in the N-junction; 10 mM succinate to record the OXPHOS capacity state FNS with convergent input of electrons via complexes I and II into the respiratory system; 10 mM glycerol-3-phosphate to activate the glycerophosphate dehydrogenase shuttle; stepwise titration with CCCP in 0.5 μM increments as needed to determine the ETS capacity state at maximum oxygen flow; 0.5 μM rotenone for complex I inhibition. All respiratory coupling states were corrected for residual oxygen consumption (ROX), which was obtained after the addition of 2.5 μM antimycin A. Finally, 5 mM ascorbate plus 0.5 mM N,N,N′,N′-tetramethyl-p-phenylenediamine dihydrochloride (TMPD) were used as substrates to assess complex IV activity [20]. The substrates were added once the previous respirometry measurement was stabilized, about every 10 min, except for the inhibitors, which were measured 5 min after their addition.

Mitochondrial membrane integrity was verified after the addition of 10 μM cytochrome C (CytC), and changes were always lower than 10%. Oxygen concentrations (mM) and oxygen flux (pmoL/s·mL), data were processed by DatLab 7.4.0.4 software (Oroboros Instruments) and specific flux (pmoL/s·mg) data were obtained. All experiments were performed using instrumental background correction and after calibration of the polarographic oxygen sensors.

### 2.4. Western Blot Analysis

Protein extracts were obtained by homogenizing a piece of frozen tissue (~1 cm^2^) in cold lysis buffer (150 mM NaCl, 1% NP-40, 50 mM Tris, pH 8, with protease inhibitors: EDTA, EGTA, PMSF and protease inhibitor cocktail tablet (Roche Diagnostic, Hoffmann-La Roche, Basel, Switzerland)). After centrifugation, clear supernatants were collected, protein content was determined (DC Protein Assay, BioRad, Hercules, CA, USA) and the same amount of protein was boiled in Laemli sample buffer (4% SDS, 20% glycerol, 0.004% bromophenol blue, 0.125 M Tris-HCl pH 6.8, 200 mM DTT) for 5 min at 95 °C (50 μg for liver homogenates and 20 μg for isolated mitochondria). For OXPHOS antibody cocktail detection, samples were heated at 50 °C for 5 min. Samples were loaded onto 8, 10, or 12% SDS- polyacrylamide electrophoresis gel (SDS-PAGE), transferred to PVDF membranes (Merck-Millipore, Burlington, MA, USA) for 1 h with cold transfer buffer (20% methanol), and blots were blocked with blocking solution (TBS and 5% milk) for 1 h at room temperature. The blots were incubated overnight at 4 °C with the primary antibodies (Table S1). After incubation with corresponding horseradish peroxidase conjugated secondary antibodies (Table S1) for 45 min at room temperature, the blots were developed with NZY Advanced ECL (NZYTech, Lisbon, Portugal). Images were taken on a luminescent image analyzer (LAS 4000 mini, GE Healthcare, Chicago, IL, USA), and densitometric analysis of the bands was performed with Image Studio Lite (version 5.2.5, Li-cor, Lincoln, NE, USA) software and expressed in arbitrary units. Band densities of target proteins were quantified and normalized with vinculin for liver homogenate samples or CytC for mitochondrial samples as loading controls.

### 2.5. Mitochondrial Membrane Potential (ΔΨm) Measurement

After animal sacrifice, a small liver lobe was removed, washed in PBS and minced with scissors. The small pieces were placed in a GentleMACS tube (Miltenyi Biotec, Bergisch Gladbach, Germany) containing 5 mL of pre-warmed DMEM with 0.75 mg/mL collagenase A. Liver pieces were homogenized in the Octo Dissociator following a pre-set program (37C_m_LIDK_1) (Miltenyi Biotec, Bergisch Gladbach, Germany), then filtered in a 100 μm filter and the collagenase reaction was stopped with 5 mL of cold DMEM. The homogenate was centrifuged and the cell pellet was washed with PBS once, and then resuspended in PBS. A 200 μL aliquot of cell suspension was treated with 10 μM cyclosporin H (1 mM stock in DMSO), labeled with 200 nM TMRM (20 μM stock, Invitrogen, Thermo Fisher Scientific, Waltham, MA, USA), and incubated at 37 °C for 30 min in the dark. After that time, 50 μL of cell suspension were passed through a flow cytometer (MACS Quant, Miltenyi Biotec, Bergisch Gladbach, Germany) to determine the TMRM labeling (20.000 events analyzed).

### 2.6. Transmission Electron Microscopy (TEM)

For TEM, embedding in epoxy resin was performed by fixing the livers with 2% glutaraldehyde (Electron Microscopy Sciences), in 0.1 M sodium cacodylate at pH 7.2, washed with cacodylate buffer containing 0.1 M sucrose, and postfixed with 1% osmium tetroxide in phosphate buffer. After washing with water and dehydration with ethanol, the samples were embedded in epoxy resin. Ultrathin slides (60 nm) were finally stained with 2% uranyl acetate prior to TEM visualization using a HITACHI HT7800 120Kv microscope at 60 Kv. Images were acquired using an EMSIS XAROSA digital camera with Olympus image analysis software. Quantifications were performed at 12 k or 25 k magnification. An average of a minimum of five visual fields was assessed for each mouse liver. Quantifications were performed with Image J software as described in [31].

### 2.7. RNA Isolation, and Quantitative RT-PCR

Total RNA from frozen liver pieces was extracted by using TRI Reagent (Thermo Fisher Scientific, Waltham, MA, USA) following the manufacturer’s indications. RNA was reverse transcribed using a Transcriptor High Fidelity cDNA Synthesis Kit following the manufacturer’s indications (Roche Diagnostic, Hoffmann-La Roche, Basel, Switzerland). cDNA was used as a template for real-time qPCR with specific primers, and UPL probes (Table S2) and Eagle Taq Universal MMX w/ROX (Roche Diagnostic) with a QuantStudio 5 System (Thermo Fisher Scientific, Waltham, MA, USA) (Table S3). Each sample was run in duplicate and was normalized to Hprt gene as the housekeeping gene. The replicates were then averaged, and fold induction was determined using 2^−ΔΔCt^ based fold-change calculations.

### 2.8. Immunofluorescence

Immunofluorescence of the mitochondrial network was performed as described in [32] with modifications. Briefly, PFA-fixed, paraffin-embedded tissues cut to 4 μm thickness, were deparaffinized and rehydrated with two changes of xylol and decreasing concentrations of ethanol. Antigen retrieval was then performed by incubating the tissues with 0.01% Trypsin in PBS at 37 °C for 10 min and microwave heating in Tris-EDTA buffer (10 mM Tris, 0.1 mM EDTA, pH 9) for 10 min. Non-specific binding was blocked with a blocking solution (10% normal horse serum, 1% bovine serum albumin, and 0.2% Triton X-100 in PBS) for 1 h. Tissues were incubated overnight at 4 °C with rabbit anti-HSP60 primary antibody (Abcam, ab46798), at 10 mg/mL in blocking solution. On the other day, tissues were washed, incubated with goat anti-rabbit conjugated to Alexa-546 secondary antibody (A-11010 Invitrogen) diluted in blocking solution for 45 h in dark and then the nuclei were stained with Hoescht 33342 (at 160 μM in PBS) for 10 min. The slides were mounted with Prolong (Invitrogen, Thermo Fisher Scientific, Waltham, MA, USA) and imaged with a confocal microscope (SP-8, Leica Microsystems, Wetzlar, Germany).

### 2.9. Statistics

Data are expressed as means ± standard deviation (S.D). The sample size (N) is indicated in each experiment. All the variables in the samples have a normal distribution, therefore, the statistical significance was tested by *t*-test when comparing two variables (*Wt* to hCOX-2- Tg) or one-way ANOVA, followed by Tukey’s posthoc test when comparing four variables (*Wt* Sham, *hCOX-2-Tg* Sham, *Wt* I/R and *hCOX-2-Tg* I/R). Analysis was performed using the statistical software SPSS (IBM Corp. Released 2020. IBM SPSS Statistics for Windows, Version 27.0., IBM Corp, Armonk, NY, USA). * *p* < 0.05, ** *p* < 0.01, *** *p* <0.001.

## 3. Results

### 3.1. Mitochondrial Respiration Is Higher When COX-2 Is Overexpressed after I/R

As a first step toward understanding the mitochondrial effects of the specific expression of cyclooxygenase 2 (COX-2) in hepatocytes during ischemia-reperfusion (I/R) injury (IRI), male *h-COX-2 Tg* mice aged 8 to 10 weeks and their Wild-type (*Wt*) siblings were subjected to 90 min of segmental (70%) warm hepatic ischemia followed by 4 h of reperfusion. IRI was associated with increased plasma alanine transaminase (ALT) and worse liver histology injury scores in *Wt* mice compared to *h-COX-2 Tg*, as described previously (Figure S1) [28].

**Figure 1:**
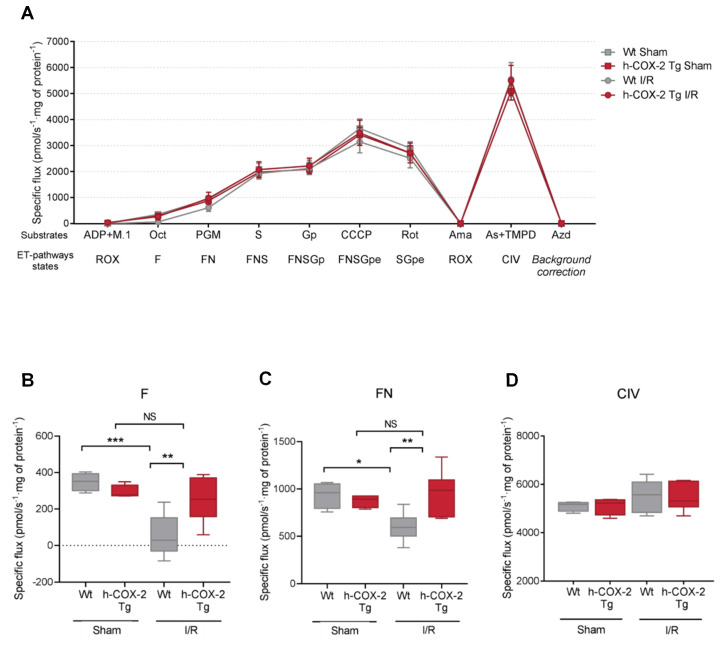
High-resolution respirometry of liver-derived mitochondria after Sham or I/R surgery of *Wt* and *h-COX-2 Tg* mice. (**A**) Complete profile of the SUIT2 protocol, with the different substrates administrated (ADP, malate (M), octanoyl-carnitine (Oct), pyruvate (P), malate, glutamate (G), succinate (S), glycerol-phosphate (Gp), CCCP and rotenone (Rot), and the ET-pathways states (ROX, residual oxygen consumption; F, F-junction pathway; N, N-junction pathway; S, succinate pathway; Gp, glycerol-phosphate pathway; e, uncoupled state, maximal respiration). After the addition of complex I inhibitor rotenone, the system was inhibited by the addition of complex III inhibitor antimycin A (Ama), and complex IV was fed with ascorbate (As) and TMPD. Later, all the system was inhibited by sodium azide and complex IV was measured after chemical background calibration. Specific flow is expressed as pmoL/s·mg of protein of states F (**B**), FN (**C**) and CIV (**D**) in detail. Box plots of 4–9 independent measures. Statistical test: one-way ANOVA, followed by Tukey’s posthoc test. * *p* < 0.05, ** *p* < 0.01; *** *p* < 0.001; NS, not significant.

The efficacy of the liver mitochondrial electron transport chain (ETC) was then assessed through high-resolution respirometry. Representative traces of mitochondria respiration are shown in Figure 1. To do so we performed high-resolution respirometry assays with mitochondria isolated from *Wt* and *h-COX-2 Tg* mice after I/R and Sham surgery. We followed a general protocol (RP2) to assess all ETC complexes and the contribution of multiple substrates to each complex. The response of all the complexes after injection of their specific substrates was very similar between mitochondria derived from *Wt* and *h-COX-2 Tg* mice (Figure 1A), but not in all pathways. Mitochondria derived from *Wt* mice after I/R had a lower respiration rate when substrates feeding the F- and N- pathways of the assay were added. If we look closely, when L-octanoylcarnitine is added to activate fatty acid oxidation and usage, *Wt* I/R mitochondria responded at a lower level, compared to *h-COX-2 Tg* derived mitochondria that behave like Sham (Figure 1B). The same profile is observed after the addition of N-pathway substrates (malate, pyruvate, and glutamate), where *h-COX-2 Tg*-derived mitochondria behave as if they are not negatively affected by I/R (Figure 1C). All other substrates, which specifically feed the other pathways, such as complex II and complex III, appeared to have the same impact on the different isolated mitochondria, and complex IV was also unaffected (Figure 1D). The differences observed in the F and N pathways are not a consequence of the variations in NAD/NADH or the total levels of AMP and ATP (Figure S2). Taken together, these observations indicate improved complex I activation in *h-COX-2 Tg* mice after I/R.

### 3.2. Higher Mitochondrial Activity Is Not Due to an Increased Presence of ETC Complexes or to a Better Association in Supercomplexes

**Figure 2:**
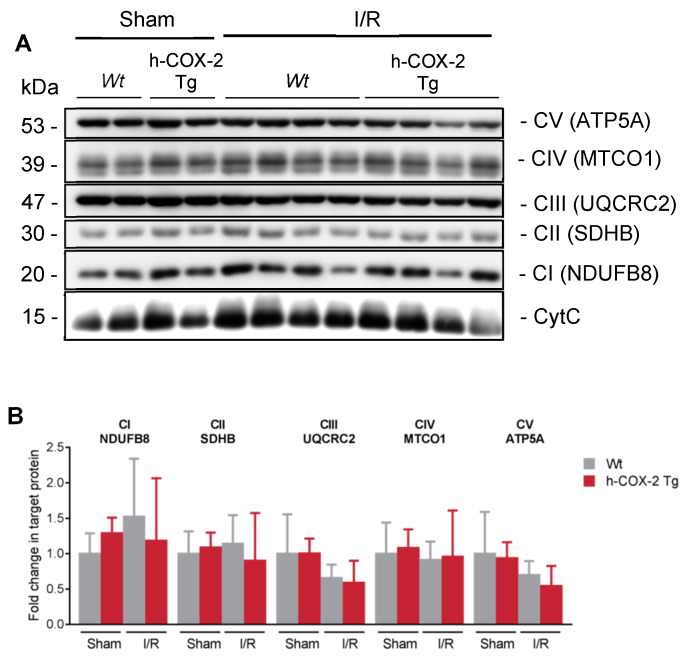
Analysis of the protein content of the electron transport chain (ETC) in liver-isolated mitochondria from *Wt* and *h-COX-2 Tg* mice after Sham or I/R surgery. (**A**) Representative western blot of one subunit of each mitochondrial ETC complex (ATP5a for CV, MTCO1 for CIV, UQCRC2 for CIII, SDHB for CII and NDUF8 for CI). Cytochrome C (CytC) was used as a loading control. (**B**) Densitometry analysis of the OXPHOS Western Blot. Bars are means ± SD of 4–9 independent measures. Statistical test: one-way ANOVA, followed by Tukey’s posthoc test.

After observing a lower rate of respiration in *Wt*-derived mitochondria after I/R, mainly due to a poorer use of the substrates feeding complex I, we thought that perhaps complex I could be affected in its presence or expression. Analyzing the expression of all ETC complexes by Western Blot analysis, we observed no differences in any of them individually between our four conditions (Figure 2). To test the supramolecular organization of these ETC proteins, we analyzed the assembly of the complexes into supercomplexes (SC) [33]. The formation of these SC was analyzed with Blue-Native Page gels (BN-PAGE), and the results showed a trend in the preservation of the stability of the complex I as suggested by the activity in respirometry (Figure S3). Taken together, these results show that the increased rate of respiration in F- and N-pathways is not due to an increased presence of complex I but to the increased stability of the SC that comprises it.

### 3.3. h-COX-2 Tg-Derived Mitochondria Have an Intact Mitochondrial Membrane Potential (ΔΨm) after I/R Compared to Wt

**Figure 3:**
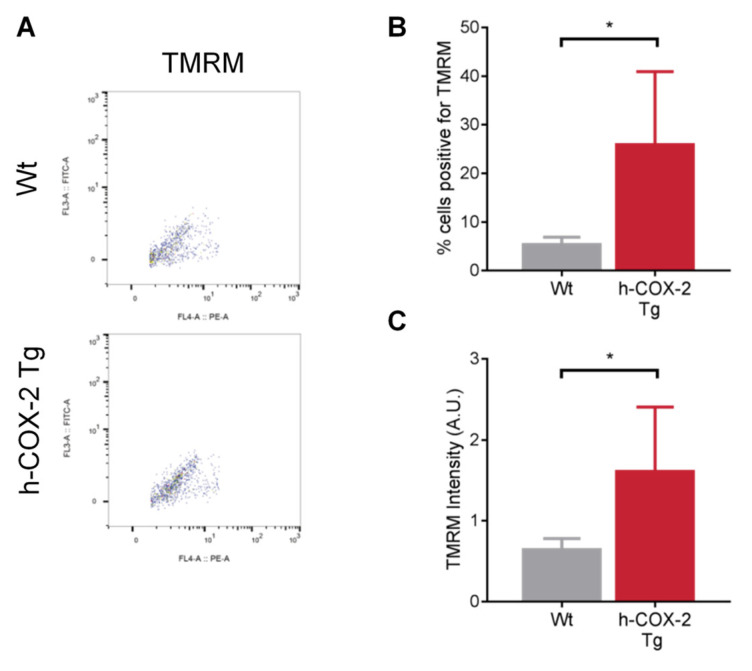
Assessment of mitochondrial membrane potential (ΔΨm) status in liver homogenates from *Wt* and *h-COX-2 Tg* mice after I/R surgery. (**A**) Representative flow cytometry images of TMRM-labelled cells. Quantification of the percentage of TMRM-positive cells (**B**) and TMRM intensity (**C**). Bars are means ± SD of five to eight independent measures. Statistical test: Student’s *t*-test. * *p* < 0.05.

The mitochondrial ETC is the main generator of mitochondrial membrane potential (ΔΨm). To assess the ΔΨm in our samples, we homogenized livers from *Wt* and *h-COX-2 Tg* mice after I/R and labeled their mitochondria with the TMRM probe. TMRM labels all positively charged mitochondria, i.e., all mitochondria that have a correct gradient potential. Conversely, mitochondria that have lost this potential will not be labeled with the probe These samples were passed through a flow cytometer (Figure 3A) and the results show that *Wt*-derived mitochondria have a lost ΔΨm, as shown by the percentage of TMRM-positive cells (Figure 3B) and the intensity of this dye (Figure 3C). In contrast, *h-COX-2 Tg*-derived mitochondria have an intact ΔΨm, (Figure 3B,C). The dissipation of ΔΨm observed in the mitochondria of *Wt* animals could be related to a higher ability of mitochondria from transgenic mice to preserve coupling after I/R, in line with a higher complex I activity, as observed by respirometric analysis.

### 3.4. Mitochondrial Aspect and Functionality Are Not Altered Because of the Overexpression of COX-2 after I/R

**Figure 4:**
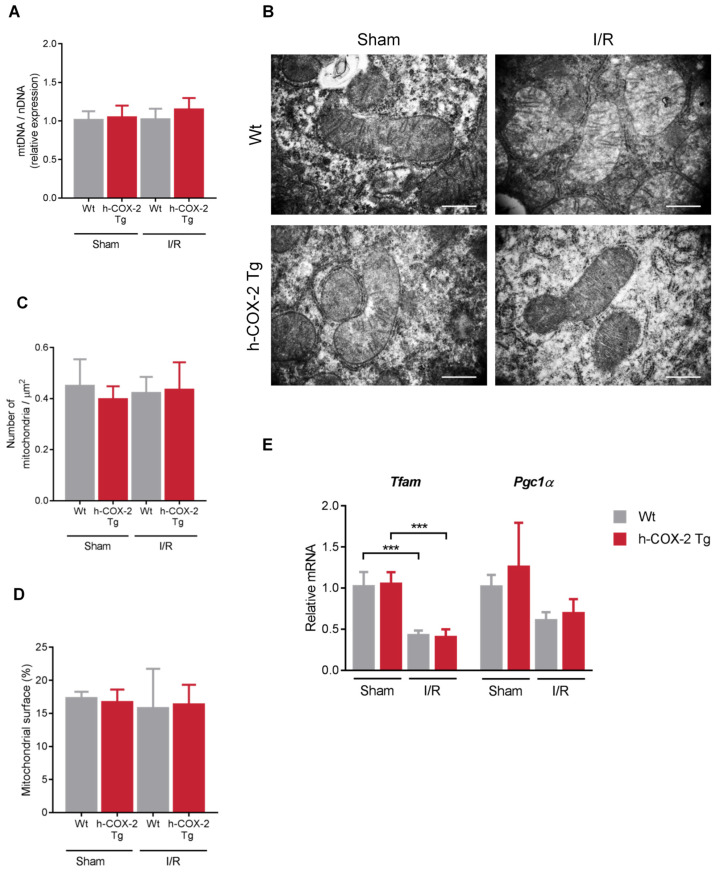
Study of the number and distribution of mitochondria in liver tissue after Sham or I/R surgery in the liver of *Wt* and *h-COX-2 Tg* mice. (**A**) Copy number of mitochondrial DNA (*CytB*) relative to nuclear DNA (*ApoB*) assessed by qPCR. Bars are means ± SD of 4–9 independent measurements. (**B**) Representative transmission electron microscopy images of liver sections from the four experimental groups. Original magnification ×25k. Scale bar: 500 nm. (**C**) Number of mitochondria relative to the image area and (**D**) relative surface area occupied by mitochondria. Bars are means ± SD of 5 images per animal, from 3–5 animals per condition. (**E**) Hepatic mRNA levels of mitochondrial biogenesis genes (*Tfam* and *Pgc1α*) in Sham and I/R conditions. Bars are means ± SD of 4 independent measurements. Statistical test: one-way ANOVA, followed by Tukey’s post-hoc test. *** *p* < 0.001.

**Figure 5:**
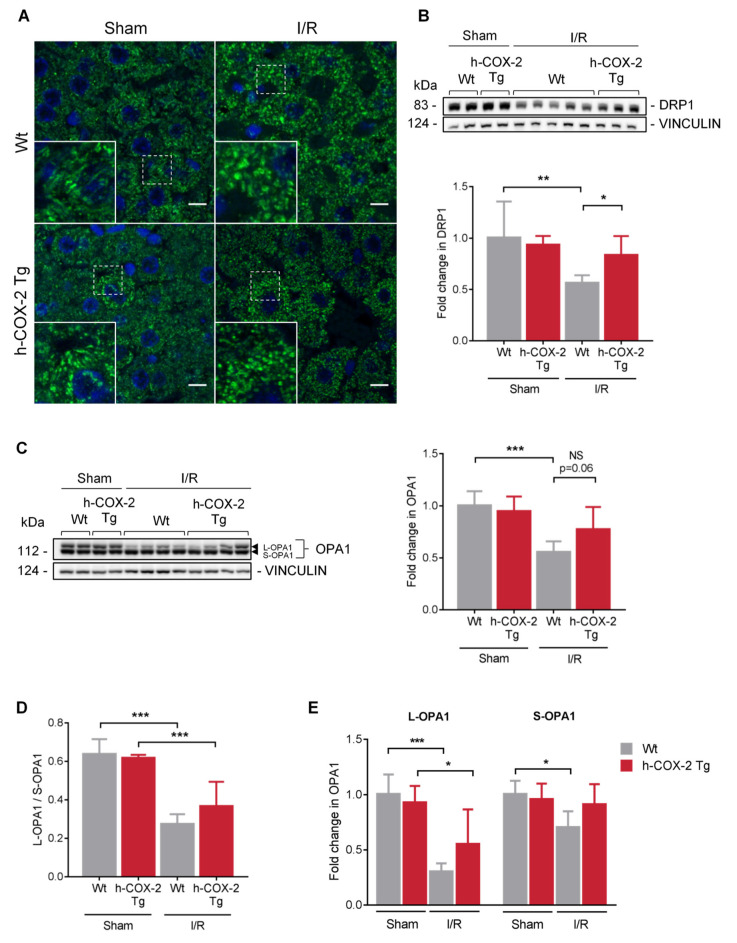
Evaluation of mitochondrial dynamics in *Wt* and *h-COX-2 Tg* liver homogenates after Sham or I/R surgery. (**A**) Representative immunofluorescence images of the mitochondrial network in the liver of the four experimental groups. Nuclei in blue (Hoescht) and mitochondria in green (HSP60). Images were taken at 63×. Scale bar: 10 μm. (**B**) Representative Western Blot and densitometry analysis of DRP1. (**C**) Representative Western Blot and densitometry analysis of OPA1. (**D**) Ratio between L-OPA1 and S-OPA1. (**E**) Differential densitometry analysis of S-OPA1 and L-OPA1. Bars are means ± SD of 4–9 independent measurements. Statistical test: one-way ANOVA, followed by Tukey’s posthoc test. * *p* < 0.05, ** *p* < 0.01; *** *p* < 0.001; NS, not significant.

Mitochondria are highly dynamic organelles and change their presence as well as their morphology to respond to cellular stress levels. Therefore, we wanted to assess the overall state of the mitochondrial network after the I/R-generated damage. Regarding the total number of mitochondria, we observed no differences when analyzing mitochondrial DNA copy number (Figure 4A), neither by I/R nor by COX-2 overexpression. We were also unable to observe differences when analyzing the number of mitochondria and their distribution in the tissue (Figure 4B–D), nor in their size (Figure S4A,B). The study was completed with the analysis of the expression of key transcription factors in mitochondrial biogenesis, the mitochondrial transcriptional factor A (*Tfam*), which stimulates mtDNA transcription, and peroxisome proliferator activated receptor coactivator-1 (*Pgc1α*), a stimulator of mitochondrial biogenesis in mammals. As described above, we detected a decrease in *Tfam* and *Pgc-1α* expression after I/R [34]. However, there are no differences between *Wt* and *h-COX-2 Tg* mice, which is in agreement with the data on mitochondrial DNA levels and the number of mitochondria evaluated by electron microscopy (Figure 4E). In addition, we performed a further study of mitochondrial cristae as an indicator of the functional status of mitochondria. Here, we also did not observe any change in the number of cristae or in the space occupied by these cristae in the mitochondria (Figure S4C–E) when comparing *Wt* and *h-COX-2 Tg* tissues after I/R or Sham surgery. What we observed was a change in the mitochondrial network due to I/R, as seen in mitochondrial immunofluorescence staining. While this network has a more filamentous appearance in the Sham samples, in I/R samples the mitochondria have a more rounded and fragmented appearance. Still, we did not detect any differences associated with h-COX-2 expression (Figure 5A).

### 3.5. OPA1 Processing Is Altered through OMA1 Activity in Wt Mice after I/R

**Figure 6:**
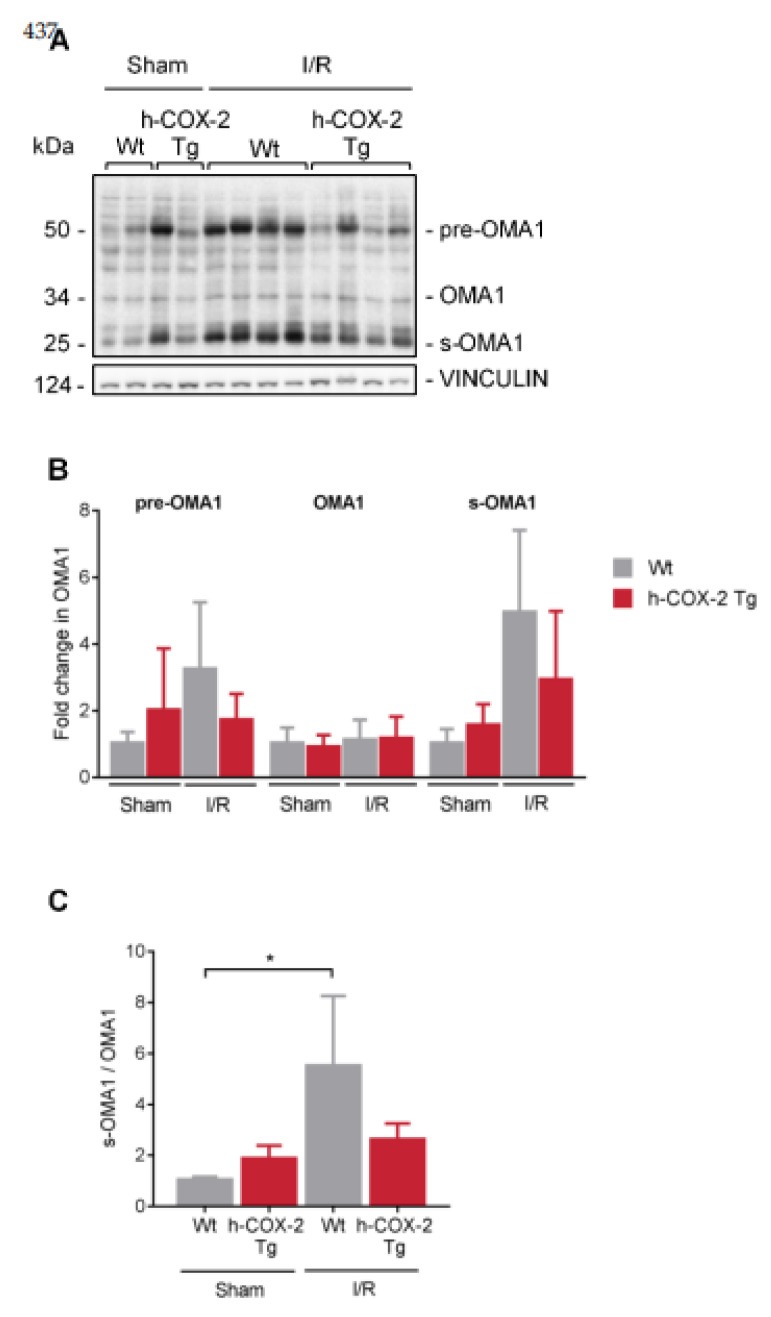
Analysis of OMA1 expression and activity. (**A**) Representative Western Blot of OMA1. (**B**) Densitometry quantification of the different forms of OMA1: pre-OMA1, OMA1 (mature form) and s-OMA1 (short form). (**C**) Ratio between the short form (s-OMA1) and the mature form (OMA1). Bars are means ± SD of three to nine independent measures. Statistical test: one-way ANOVA, followed by Tukey’s posthoc test. * *p* < 0.05.

To establish a link between COX-2 and mitochondrial dynamics as a possible protective molecular mechanism, we have analyzed different proteins involved in fusion-fission mechanisms to better understand the state of mitochondria in our model. Fission appears to be challenged in *Wt*-derived mitochondria, as we observed a decrease in DRP1 levels after I/R. These levels are somewhat maintained in *h-COX-2 Tg*-derived mitochondria (Figure 5B). However, we observed no further differences in other fission-related proteins (Figure S5A). Interestingly, although MFN1 and MFN2, are not altered after I/R (Figure S5B,C), a decrease in OPA1 is observed in *Wt* animals after IRI (Figure 5C). When analyzing OPA1 expression in-depth, we observed a reduced ratio of L-/S-OPA1 forms (Figure 5D) due to a prominent loss of OPA1 long forms after I/R in *Wt* livers, which was instead almost fully prevented in *h-COX-2 Tg* animals (Figure 5E). We also analyzed the presence of the two IMM proteases that control this processing. While YME1L1 shows no differences (Figure S5D), OMA1 has a higher presence of its short form corresponding to its active form in *Wt* livers compared to *h-COX-2 Tg* after I/R, differences that are not observed in Sham (Figure 6). Taken together, our findings suggest that h-COX-2 regulates IRI by modulating OPA1 processing through OMA1.

## 4. Discussion

**Figure 7:**
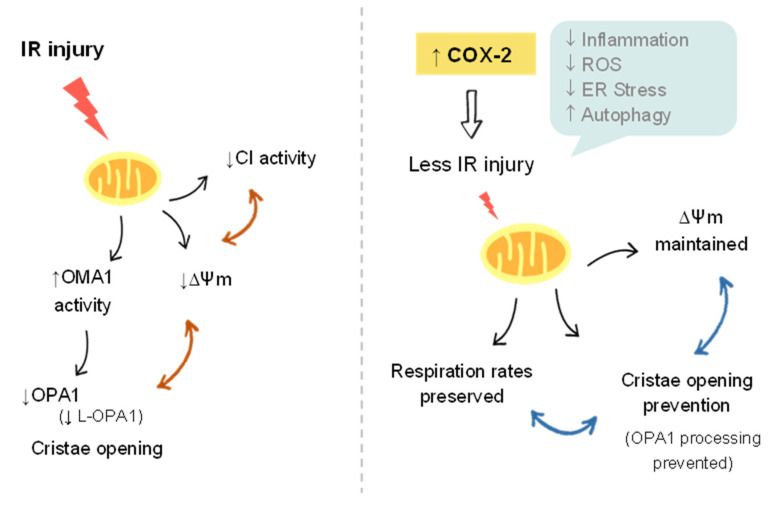
Model of mitochondrial damage after I/R. Comparison between *Wt* and *h-COX-2 Tg* livers. In *Wt* conditions, I/R injury directly impacts mitochondria, decreasing mitochondrial membrane potential (ΔΨm), affecting complex I activity and activating the enzyme OMA1. OMA1 processes OPA1, leading to a reduced presence of the long form of OPA1 (L-OPA1), causing a destabilization of the cristae and leading to their opening. In the presence of h-COX-2, I/R injury in the livers is reduced, therefore mitochondria are less affected: ΔΨm is preserved, complex I maintains its function and cristae remain tight. All these data are integrated with COX-2 protection mechanisms previously described in [28].

Hepatic ischemia-reperfusion (I/R) injury (IRI) is a major cause of morbidity and mortality in liver resection and transplantation. The mechanisms of organ damage following I/R have been widely studied, and involve the complex interactions of multiple pathways. Unfortunately, despite intensive research, effective therapeutic approaches for the prevention/treatment of IRI remain clinically limited. Our previous data support the idea of a protective effect of hepatic cyclooxygenase 2 (COX-2) induction and its subsequent increase in prostaglandins (PGs) against IRI [28]. As the deleterious effects associated with hepatic IRI are known to cause mitochondrial dysfunction, we wanted to evaluate whether the protective effects of COX-2 could be the result of changes in mitochondrial function. In the present study, we identified that mitochondria from *h-COX-2 Tg* mice have an increased respiratory capacity through fatty acid oxidation (F-pathway) using a combination of fatty acids and malate via the electron transfer flavoprotein complex (CETF). We identified this increase also through the NADH pathway (N-pathway) using a substrate combination of malate, pyruvate and glutamate, which stimulates dehydrogenases with a reduction in NAD^+^ feeding electrons into complex I. This increased respiratory capacity also manifests itself in the maintenance of the mitochondrial membrane potential (ΔΨm). While decreased DRP1 levels may facilitate an unopposed fusion, we observed no significant effects in mitochondrial elongation. We analyzed S/L forms of OPA1 and observed the maintenance of L forms consistent with the prevention of OMA1 activation and OPA1 cleavage, which otherwise occurred in Wild-type (*Wt*) under I/R (Figure 7). Conversely, and in the absence of a net pro-fusion role, S/L-OPA1 ratios may be indicative of an ultrastructural effect in the preserving cristae to facilitate the activity of respiratory (super)complexes [35], which may parallel the lower reactive oxygen species (ROS) levels in *h-COX-2 Tg* mice [28].

Mitochondrial morphology is involved in mitochondrial adaptation to specific cellular and tissue demands and influences many aspects of mitochondrial biology, including electron transport chain (ETC) activity, apoptotic sensitivity, and mitophagy [36]. We, therefore, determined whether hepatocyte-specific expression of h-COX-2 could influence the mitochondrial network. We did not observe any genotype-associated changes, although there was a change after IRI in which mitochondria have a more rounded structure (Figure 5). These doughnut-shaped mitochondria that appeared during hypoxia and are maintained during reoxygenation have already been described. Some components of the mitochondrial metabolic machinery were better preserved in doughnut-shaped mitochondria than in mitochondria that retained the linear shape. This includes the mobility of the free end of mitochondria and the increased association between IMM and OMM that could optimize the interaction between MFNs and OPA1 [37]. Thus, doughnut-shaped remodeling of mitochondria could be a component of a protective mechanism that helps to preserve organelles under conditions of metabolic stress [38].

Accumulating studies in recent years have shown that mitochondrial dynamics are closely related to mitochondrial function and homeostasis [39]. Although we did not observe any differences in the mitochondrial network, we did observe a decrease in DRP1 and OPA1 levels in liver homogenates from *Wt* animals after I/R, whereas the levels of both proteins are preserved in transgenic animals (Figure 5). DRP1 is required for mitochondria to be functionally active. Inhibition of DRP1 has been demonstrated to confer protection under various stress conditions. In the kidney, renal injury has been reported to occur through the DRP1-dependent induction of mitochondrial fragmentation and apoptosis, and prevention of this process is beneficial [40]. Moreover, the pharmacological inhibition of the mitochondrial fission protein DRP1 protected adult murine cardiomyocytes against I/R injury [41]. This cardioprotective mechanism appears to be related to an increased mitophagy process [42]. Therefore, the decreased DRP1 levels shown in *Wt* animals may reflect the attempt of the *Wt* animal mitochondria to protect themselves from injury. Further investigation is required to examine this possibility.

OPA1-mediated mitochondrial protection in organ reperfusion injury has been verified in cardiac and brain I/R injury models [43,44,45]. Although, as expected, OPA1 levels decreased after I/R, h-COX-2 expression attenuated this decrease. A protective mechanism against renal damage due to I/R has also been associated with an OPA1-mediated enhancement of mitochondrial fusion and the preservation of cristae structure [45,46]. Furthermore, as detected in our model, the protective mechanism was not due to variations in MFN1 or MFN2 levels [47].

Under normal conditions, proteolytic cleavage of long isoforms of OPA1 (L-OPA1) results in a balanced accumulation of L-OPA1 and short forms of OPA1 (S-OPA1) [17,18,48]. In our model, we detected increased processing of OPA1 in *Wt* animals that may be associated with loss of ΔΨm in *Wt* animals after I/R. Loss of ΔΨm triggers OPA1 proteolysis and inhibits mitochondrial fusion [17,48]. Depolarization of the ΔΨm causes the processing of L-OPA1 in an OMA1-dependent manner, suggesting that OMA1 regulates L-OPA1 stability in response to the bioenergetic state of mitochondria in the cell [49,50,51]. Consistent with these data, we did not observe changes in YME1L levels in either *Wt* or Tg animals, whereas our results identify OMA1-mediated OPA1 processing as a general cellular response following IRI (Figure 6).

L-OPA1 cleavage by OMA1 is enhanced by mitochondrial dysfunction including loss of ΔΨm, reduced respiration rates, and oxidative phosphorylation (OXPHOS) [49,50]. These data are consistent with our results showing increased OXPHOS with specific NADH-linked substrates (Figure 1). The activity of complex I of the mitochondrial respiratory chain has been associated with liver protection after IRI. Thus, the *Alr* gene protects mice from normal hepatic IRI, primarily by increasing mitochondrial respiratory chain complex I activity, alleviating mitochondrial swelling, preserving mitochondrial ultrastructure and inhibiting cytochrome C leakage [47]. Animals pretreated with the mitochondrial antioxidant mitoquinone (MitoQ) attenuated I/R-induced liver dysfunction and damage in part due to a significant enhancement of complex I activity, but without changes in complex II and IV activities, in agreement with our data.

Cristae remodeling induced by changes in the L-OPA1 metabolism can affect the structural assembly and function of inner mitochondrial membrane (IMM) proteins, in particular, respiratory chain supercomplexes (SC) [52]. It is now well established that the individual respiratory complexes can be organized into SC which provides an advantage in optimizing metabolic resources. [53]. Therefore, we assessed whether the structural organization of the mitochondrial respiratory chain differed between *Wt* and *h-COX-2 Tg* mice after Sham or IRI conditions. Complex I was present both alone and bound to high molecular weight supercomplexes. The composition of these SC was confirmed by the electrotransfer of the proteins to PVDF membranes followed by immunoblotting against a subunit of complex I (NDUFA5). Interpretation of BN-PAGE data points to the role of L-OPA1 in stabilizing SC organization as responsible for the differences in mitochondrial OXPHOS function detected in *h-COX-2 Tg* mice (Figure S3).

Electron transport by the different complexes of the ETC and the generation of mitochondrial ΔΨm are key for ATP synthesis [54]. Mitochondria from transgenic mice showed, in addition to an increase in complex I activity by respirometry, an expected preservation in ΔΨm, but, strikingly, only a trend of increase in ATP and AMP levels (Figure S2). Ischemic preconditioning (IP), which increases endogenous COX-2 expression, increases hepatic ATP content probably as a result of decreased ATP use rather than ATP production or consumption [55]. Following IRI, an increased phosphorylated adenosine monophosphate–activated protein kinase (pAMPKα)/AMPKα ratio was observed in *h-COX-2 Tg* compared to *Wt* mice [28]. On the other hand, COX-2 overexpression increased energy expenditure, protecting mice from diet-induced obesity, suggesting that the prostaglandin pathway regulates systemic energy homeostasis [56]. Considering all these data, we can speculate that the same increase in ATP and AMP levels observed after IRI may be due to the dual action of COX-2 to preserve ATP consumption under stress and modulate thermogenesis and systemic energy expenditure.

Recent studies show that the stabilization of L-OPA1 reduced the exorbitant generation of ROS induced by cerebral I/R by increasing the activity of antioxidant components, such as SOD, GPX, and the GSH/GSSG ratio. These results suggest that L-OPA1 may act as an attractive target for quenching oxidative stress-induced neuronal damage after I/R [57]. Thus, the increase in L-OPA1 levels detected could be related to our previous results showed a clear induction of *Nrf2*, *Hmox1*, *Sod1*, and *Sod2* expression in *h-COX-2 Tg* mice after IRI compared to *Wt* [28]. We analyzed mitochondrial ROS production in our model and, in line with the idea set out above, we did not observe changes in ROS production despite the enhancement of complex I activity observed in the *h-COX-2 Tg* mice (Figure S6).

OPA1 has been shown to play a role in multiple forms of cell death in vivo. Thus, mild overexpression of OPA1 protects mice from multiple forms of damage, including cristae remodeling in myocardial and cerebral infarction, hepatocellular apoptosis and muscle atrophy [45]. We found an anti-apoptotic effect of COX-2 by an attenuated response in the BAX/BCL-2 ratio and caspase 3 activity in *h-COX-2 Tg* mice after I/R [28]. Our data would be in agreement with previous results that have shown that OMA1 depletion and L-OPA1 stabilization can provide resistance to cytochrome C release and cell death independently of the restoration of cristae morphology in vivo [58]. Notably, overexpression of OPA1 protects against Fas-mediated hepatocellular apoptosis [45]. Our previous data showed that *h-COX-2 Tg* mice were resistant to Fas-mediated apoptosis [24], suggesting an anti-apoptotic role of COX-2-dependent PGs through the modulation of OPA1 processing.

## 5. Conclusions

Our data demonstrated that COX-2 plays an essential protective role in liver injury, in part by preserving mitochondrial functionality, increasing respiratory capacity at the complex I level and maintaining mitochondrial membrane potential. This effect could be by inhibiting mitochondrial fission and preserving mitochondrial ultrastructure through the OMA1-regulated processing of OPA1.

## Data Availability

Information on all the westerns blots in the study are included in Supplementary Materials.

## Supplementary Materials

The following supporting information can be downloaded at: https://www.mdpi.com/article/10.3390/antiox11091724/s1, Supplementary Tables S1–S3, Supplementary Figures S1–S6, and Supplementary Materials and Methods.

## References

[1] Barahman M., Asp P., Roy-Chowdhury N., Kinkhabwala M., Roy-Chowdhury J., Kabarriti R., Guha C. (2019). Hepatocyte Transplantation: Quo Vadis?. Int. J. Radiat. Oncol. Biol. Phys..

[2] Jaeschke H. (2003). Molecular Mechanisms of Hepatic Ischemia-Reperfusion Injury and Preconditioning. Am. J. Physiol. Liver Physiol..

[3] Coito J., Duarte S., Moore C., Busuttil R.W., Hamada A.T., Tsuchihashi S., Avanesyan A. (2008). Injury Recruitment in Hepatic Ischemia/Reperfusion Immune Responses and Impairs Neutrophil Cyclooxygenase-2 Deficiency Enhances Th2. J. Immunol. Ref..

[4] Demiryilmaz I., Turan M.I., Kisaoglu A., Gulapoglu M., Yilmaz I., Suleyman H. (2014). Protective Effect of Nimesulide against Hepatic Ischemia/Reperfusion Injury in Rats: Effects on Oxidant/Antioxidants, DNA Mutation and COX-1/COX-2 Levels. Pharmacol. Rep..

[5] Guzy R.D., Schumacker P.T. (2006). Oxygen Sensing by Mitochondria at Complex III: The Paradox of Increased Reactive Oxygen Species during Hypoxia. Exp. Physiol..

[6] Klinman J.P. (2007). How Do Enzymes Activate Oxygen without Inactivating Themselves?. Acc. Chem. Res..

[7] Selzner N. (2003). Protective Strategies against Ischemic Injury of the Liver. Gastroenterology.

[8] Rolo A.P., Teodoro J.S., Peralta C., Rosello-Catafau J., Palmeira C.M. (2009). Prevention of I/R Injury in Fatty Livers by Ischemic Preconditioning Is Associated with Increased Mitochondrial Tolerance: The Key Role of ATPsynthase and Mitochondrial Permeability Transition. Transpl. Int..

[9] Kalogeris T., Bao Y., Korthuis R.J. (2014). Mitochondrial Reactive Oxygen Species: A Double Edged Sword in Ischemia/Reperfusion vs Preconditioning. Redox Biol..

[10] Giacomello M., Pyakurel A., Glytsou C., Scorrano L. (2020). The Cell Biology of Mitochondrial Membrane Dynamics. Nat. Rev. Mol. Cell Biol..

[11] MacVicar T., Langer T. (2016). OPA1 Processing in Cell Death and Disease—The Long and Short of It. J. Cell Sci..

[12] Frezza C., Cipolat S., Martins de Brito O., Micaroni M., Beznoussenko G.V., Rudka T., Bartoli D., Polishuck R.S., Danial N.N., De Strooper B. (2006). OPA1 Controls Apoptotic Cristae Remodeling Independently from Mitochondrial Fusion. Cell.

[13] Detmer S.A., Chan D.C. (2007). Functions and Dysfunctions of Mitochondrial Dynamics. Nat. Rev. Mol. Cell Biol..

[14] Cipolat S., De Brito O.M., Dal Zilio B., Scorrano L. (2004). OPA1 Requires Mitofusin 1 to Promote Mitochondrial Fusion. Proc. Natl. Acad. Sci. USA.

[15] Pagliuso A., Cossart P., Stavru F. (2018). The Ever-Growing Complexity of the Mitochondrial Fission Machinery. Cell. Mol. Life Sci..

[16] Yu R., Jin S., Lendahl U., Nistér M., Zhao J. (2019). Human Fis1 Regulates Mitochondrial Dynamics through Inhibition of the Fusion Machinery. EMBO J..

[17] Griparic L., Kanazawa T., Van Der Bliek A.M. (2007). Regulation of the Mitochondrial Dynamin-like Protein Opa1 by Proteolytic Cleavage. J. Cell Biol..

[18] Song Z., Chen H., Fiket M., Alexander C., Chan D.C. (2007). OPA1 Processing Controls Mitochondrial Fusion and Is Regulated by MRNA Splicing, Membrane Potential, and Yme1L. J. Cell Biol..

[19] Stiburek L., Cesnekova J., Kostkova O., Fornuskova D., Vinsova K., Wenchich L., Houstek J., Zeman J. (2012). YME1L Controls the Accumulation of Respiratory Chain Subunits and Is Required for Apoptotic Resistance, Cristae Morphogenesis, and Cell Proliferation. Mol. Biol. Cell.

[20] Gnaiger E. (2020). Mitochondrial Pathways and Respiratory Control. Bioenerg. Commun..

[21] Davila M.P., Muñoz P.M., Tapia J.A., Ferrusola C.O., Da Silva C.C.B., Peña F.J. (2015). Inhibition of Mitochondrial Complex I Leads to Decreased Motility and Membrane Integrity Related to Increased Hydrogen Peroxide and Reduced ATP Production, While the Inhibition of Glycolysis Has Less Impact on Sperm Motility. PLoS ONE.

[22] Callejas N.A., Boscá L., Williams C.S., DuBois R.N., Martín-Sanz P. (2000). Regulation of Cyclooxygenase 2 Expression in Hepatocytes by CCAAT/Enhancer-Binding Proteins. Gastroenterology.

[23] Martín-Sanz P., Callejas N.A., Casado M., Díaz-Guerra M.J.M., Boscá L. (1998). Expression of Cyclooxygenase-2 in Foetal Rat Hepatocytes Stimulated with Lipopolysaccharide and pro-Inflammatory Cytokines. Br. J. Pharmacol..

[24] Casado M., Mollá B., Roy R., Fernández-Martínez A., Cucarella C., Mayoral R., Boscá L., Martín-Sanz P. (2007). Protection against Fas-Induced Liver Apoptosis in Transgenic Mice Expressing Cyclooxygenase 2 in Hepatocytes. Hepatology.

[25] Krause M.M., Brand M.D., Krauss S., Meisel C., Vergin H., Burmester G.-R., Buttgereit F. (2003). Nonsteroidal Antiinflammatory Drugs and a Selective Cyclooxygenase 2 Inhibitor Uncouple Mitochondria in Intact Cells. Arthritis Rheum..

[26] Fu H., Chen H., Wang C., Xu H., Liu F., Guo M., Wang Q., Shi X. (2012). Flurbiprofen, a Cyclooxygenase Inhibitor, Protects Mice from Hepatic Ischemia/Reperfusion Injury by Inhibiting GSK-3β Signaling and Mitochondrial Permeability Transition. Mol. Med..

[27] Tolba R.H., Fet N., Yonezawa K., Taura K., Nakajima A., Hata K., Okamura Y., Uchinami H., Klinge U., Minor T. (2014). Role of Preferential Cyclooxygenase-2 Inhibition by Meloxicam in Ischemia/Reperfusion Injury of the Rat Liver. Eur. Surg. Res..

[28] Motiño O., Francés D.E., Casanova N., Fuertes-Agudo M., Cucarella C., Flores J.M., Vallejo-Cremades M.T., Olmedilla L., Pérez Peña J., Bañares R. (2019). Protective Role of Hepatocyte Cyclooxygenase-2 Expression against Liver Ischemia–Reperfusion Injury in Mice. Hepatology.

[29] Frezza C., Cipolat S., Scorrano L. (2007). Organelle Isolation: Functional Mitochondria from Mouse Liver, Muscle and Cultured Filroblasts. Nat. Protoc..

[30] Doerrier C., Garcia-Souza L.F., Krumschnabel G., Wohlfarter Y., Mészáros A.T., Gnaiger E. (2018). High-Resolution FluoRespirometry and OXPHOS Protocols for Human Cells, Permeabilized Fibers from Small Biopsies of Muscle, and Isolated Mitochondria. Methods Mol. Biol..

[31] Lam J., Katti P., Biete M., Mungai M., AshShareef S., Neikirk K., Garza Lopez E., Vue Z., Christensen T.A., Beasley H.K. (2021). A Universal Approach to Analyzing Transmission Electron Microscopy with ImageJ. Cells.

[32] van Zutphen T., Ciapaite J., Bloks V.W., Ackereley C., Gerding A., Jurdzinski A., de Moraes R.A., Zhang L., Wolters J.C., Bischoff R. (2016). Malnutrition-Associated Liver Steatosis and ATP Depletion Is Caused by Peroxisomal and Mitochondrial Dysfunction. J. Hepatol..

[33] Schägger H. (2001). Respiratory Chain Supercomplexes. IUBMB Life Int. Union Biochem. Mol. Biol. Life.

[34] Bi J., Zhang J., Ren Y., Du Z., Li Q., Wang Y., Wei S., Yang L., Zhang J., Liu C. (2019). Irisin Alleviates Liver Ischemia-Reperfusion Injury by Inhibiting Excessive Mitochondrial Fission, Promoting Mitochondrial Biogenesis and Decreasing Oxidative Stress. Redox Biol..

[35] Quintana-Cabrera R., Manjarrés-Raza I., Vicente-Gutiérrez C., Corrado M., Bolaños J.P., Scorrano L. (2021). Opa1 Relies on Cristae Preservation and ATP Synthase to Curtail Reactive Oxygen Species Accumulation in Mitochondria. Redox Biol..

[36] Labbé K., Murley A., Nunnari J. (2014). Determinants and Functions of Mitochondrial Behavior. Annu. Rev. Cell Dev. Biol..

[37] Zhou Y., Long Q., Wu H., Li W., Qi J., Wu Y., Xiang G., Tang H., Yang L., Chen K. (2020). Topology-Dependent, Bifurcated Mitochondrial Quality Control under Starvation. Autophagy.

[38] Liu X., Hajnó G. (2011). Altered Fusion Dynamics Underlie Unique Morphological Changes in Mitochondria during Hypoxia-Reoxygenation Stress. Cell Death Differ..

[39] Bhargava P., Schnellmann R.G. (2017). Mitochondrial Energetics in the Kidney. Nat. Rev. Nephrol..

[40] Brooks C., Wei Q., Cho S.-G., Dong Z. (2009). Regulation of Mitochondrial Dynamics in Acute Kidney Injury in Cell Culture and Rodent Models. J. Clin. Investig..

[41] Ong S.-B., Subrayan S., Lim Y.S., Yellon D.M., Davidson S.M., Hausenloy D.J. (2010). Inhibiting Mitochondrial Fission Protects the Heart Against Ischemia/Reperfusion Injury. Circulation.

[42] Bouche L., Kamel R., Tamareille S., Garcia G., Villedieu C., Pillot B., Gueguen N., Chehaitly A., Manuel Chao de la Barca J., Beaumont J. (2021). DRP1 Haploinsufficiency Attenuates Cardiac Ischemia/Reperfusion Injuries. PLoS ONE.

[43] Heckmann B.L., Tummers B., Green D.R. (2019). Crashing the Computer: Apoptosis vs. Necroptosis in Neuroinflammation. Cell Death Differ..

[44] Krause J., Löser A., Lemoine M.D., Christ T., Scherschel K., Meyer C., Blankenberg S., Zeller T., Eschenhagen T., Stenzig J. (2018). Rat Atrial Engineered Heart Tissue: A New In Vitro Model to Study Atrial Biology. Basic Res. Cardiol..

[45] Varanita T., Soriano M.E., Romanello V., Zaglia T., Quintana-Cabrera R., Semenzato M., Menabò R., Costa V., Civiletto G., Pesce P. (2015). The Opa1-Dependent Mitochondrial Cristae Remodeling Pathway Controls Atrophic, Apoptotic, and Ischemic Tissue Damage. Cell Metab..

[46] Quintana-Cabrera R., Quirin C., Glytsou C., Corrado M., Urbani A., Pellattiero A., Calvo E., Vázquez J., Enríquez J.A., Gerle C. (2018). The Cristae Modulator Optic Atrophy 1 Requires Mitochondrial ATP Synthase Oligomers to Safeguard Mitochondrial Function. Nat. Commun..

[47] Jiang S.J., Li W., An W. (2013). Adenoviral Gene Transfer of Hepatic Stimulator Substance Confers Resistance against Hepatic Ischemia-Reperfusion Injury by Improving Mitochondrial Function. Hum. Gene Ther..

[48] Ishihara N., Fujita Y., Oka T., Mihara K. (2006). Regulation of Mitochondrial Morphology through Proteolytic Cleavage of OPA1. EMBO J..

[49] Ehses S., Raschke I., Mancuso G., Bernacchia A., Geimer S., Tondera D., Martinou J.C., Westermann B., Rugarli E.I., Langer T. (2009). Regulation of OPA1 Processing and Mitochondrial Fusion by M-AAA Protease Isoenzymes and OMA1. J. Cell Biol..

[50] Head B., Griparic L., Amiri M., Gandre-Babbe S., Van Der Bliek A.M. (2009). Inducible Proteolytic Inactivation of OPA1 Mediated by the OMA1 Protease in Mammalian Cells. J. Cell Biol..

[51] Quirós P.M., Ramsay A.J., Sala D., Fernández-Vizarra E., Rodríguez F., Peinado J.R., Fernández-García M.S., Vega J.A., Enríquez J.A., Zorzano A. (2012). Loss of Mitochondrial Protease OMA1 Alters Processing of the GTPase OPA1 and Causes Obesity and Defective Thermogenesis in Mice. EMBO J..

[52] Wittig I., Carrozzo R., Santorelli F.M., Schägger H. (2006). Supercomplexes and Subcomplexes of Mitochondrial Oxidative Phosphorylation. Biochim. Biophys. Acta Bioenerg..

[53] García-Poyatos C., Cogliati S., Calvo E., Hernansanz-Agustín P., Lagarrigue S., Magni R., Botos M., Langa X., Amati F., Vázquez J. (2020). Scaf1 Promotes Respiratory Supercomplexes and Metabolic Efficiency in Zebrafish. EMBO Rep..

[54] Zorova L.D., Popkov V.A., Plotnikov E.Y., Silachev D.N., Pevzner I.B., Jankauskas S.S., Babenko V.A., Zorov S.D., Balakireva A.V., Juhaszova M. (2018). Mitochondrial Membrane Potential. Anal. Biochem..

[55] Peralta C., Bartrons R., Serafin A., Blázquez C., Guzmán M., Prats N., Xaus C., Cutillas B., Gelpí E., Roselló-Catafau J. (2001). Adenosine Monophosphate-Activated Protein Kinase Mediates the Protective Effects of Ischemic Preconditioning on Hepatic Ischemia-Reperfusion Injury in the Rat. Hepatology.

[56] Francés D.E., Motiño O., Agrá N., González-Rodríguez Á., Fernández-Álvarez A., Cucarella C., Mayoral R., Castro-Sánchez L., García-Casarrubios E., Boscá L. (2015). Hepatic Cyclooxygenase-2 Expression Protects against Diet-Induced Steatosis, Obesity, and Insulin Resistance. Diabetes.

[57] Lai Y., Lin P., Chen M., Zhang Y., Chen J., Zheng M., Liu J., Du H., Chen R., Pan X. (2020). Restoration of L-OPA1 Alleviates Acute Ischemic Stroke Injury in Rats via Inhibiting Neuronal Apoptosis and Preserving Mitochondrial Function. Redox Biol..

[58] Cipolat S., Rudka T., Hartmann D., Costa V., Serneels L., Craessaerts K., Metzger K., Frezza C., Annaert W., D’Adamio L. (2006). Mitochondrial Rhomboid PARL Regulates Cytochrome c Release during Apoptosis via OPA1-Dependent Cristae Remodeling. Cell.

