# Supplementary material for: COX-2 Expression in Hepatocytes Improves Mitochondrial Function after Hepatic Ischemia-Reperfusion Injury
Source: Antioxidants (Basel). 2022 Aug 30;11(9):1724. doi: 10.3390/antiox11091724 (PMC9495319; doi:10.3390/antiox11091724)
Supplement: Supplementary file 1 [file antioxidants-11-01724-s001.zip › antioxidants-1848818-supplementary.pdf]

SUPPLEMENTARY DATA

Supplementary Materials and Methods

Chemicals and reagents

Antibodies were from Abcam (Cambridge, UK), Thermo Fisher Scientific (Waltham, MA USA), Proteintech (Rosemont, IL, USA). Sigma, (Merck Life Science S.L.U. (Darmstadt, Germany), and Santa Cruz Biotechnology (Dallas, TX, USA). Reagents were from Merck Life Science S.L.U, Scharlau (Barcelona, Spain), Roche Diagnostics (Basel, Switzerland), and NZYtech (Lisboa, Portugal). Reagents for electrophoresis were obtained from BioRad (Hercules, CA, USA), Merck Life Science S.L.U, Acros Organics (Thermo Fisher Scientific, Geel, Belgium), Serva (Heidelberg, Germany), TCI (Zwijndrecht, Belgium), and BD Life Sciences (Franklin Lakes, New Jersey, U.S). Reagents for respirometry were obtained from Sigma, (Merck Life Science S.L.U), Calbiochem (San Diego, CA, USA), and Tocris (Bristol, UK).

Generation and maintenance of the h-COX-2 Tg mice

The *h-COX-2-Tg* mice constitutively express human COX-2 enzyme under the control of the human APOE promoter and its specific hepatic control region (HCR). The transgene is maintained in heterozygosity. The *h-COX-2-Tg* mice and their corresponding *Wt* littermates were generated by systematically mating heterozygous B6D2JRccHsd-Tg(APOE-*PTGS2*)4/Upme males expressing 55 copies of the transgene with B6D2JRccHsd F1 female mice in our animal house for more than 7 generations. Phenotypically, *h-COX-2-Tg* mice were indistinguishable from *Wt*, with no histological changes in their livers at 12 weeks of age [24]. Mice were maintained at the IBV-CSIC facilities on a 14/10 light/dark cycle under constant temperature (23 °C) with chow (SAFE A40 Rodent Maintenance Diet, Rettenmaier Iberica, Spain), and water provided ad libitum. Animals were housed in filter-lidded cages, using poplar bedding and nest material as enrichment. All experimental procedures were performed during the light cycle.

DNA Isolation and qPCR for mitochondrial to genomic DNA ratio

Total DNA was isolated from small pieces of frozen liver tissues (~1 cm^2) after sham and ischemia/reperfusion (I/R) surgery. The pieces were homogenized in 100 μL of Buffer I (10 mM Tris-HCl, pH 7.0, 10 mM $MgCl_2$, 0.5% (v/v) Triton X-100) in a small glass-potter manually. The homogenate was transferred to a 1.5 mL tube and 5 U/μL of RNase was added. The tubes were incubated at 37 ºC for 30 min. Next, 300 μL of lysis buffer (2% (w/v) cetyl trimethyl ammonium bromide (CTAB), 100 mM Tris-HCl, pH 8.0, 20 mM EDTA, 1.4 M NaCl) plus 2% (v/v) β-mercaptoethanol was added. A brief vortex was applied and the tubes were incubated at 65 ºC for 30 min. After this time, a volume of chloroform–isoamyl alcohol (24:1) was added, the tubes were shaken until the combination of the 2 phases was achieved and centrifuged at 16000x *g* for 10 min at room temperature. The upper phase was then collected in new tubes and a volume of isopropanol was added. The tubes were kept at -20 ºC for at least 1 h and then centrifuged at 16000x *g* for 25 min at 4 ºC. The supernatant was discarded and the DNA pellet was washed twice with 70% EtOH, dried at room temperature and resuspended in 10 mM Tris/0.1 mM EDTA.

DNA quantification was performed with a Nanodrop 2000 (Thermo Fisher Scientific), and was diluted to a final concentration of 10 ng/mL. *CytB* gene as mitochondrial DNA (mtDNA) marker, and *ApoB* gene as nuclear DNA (nDNA), were amplified by qPCR with Power SYBR Green Master Mix (Thermo Fisher Scientific) with specific primers (Table S1). The qPCR was performed with a QuantStudio 5 System (Thermo Fisher Scientific) (Table S2). Each sample was run in duplicate and mtDNA was normalized to nDNA. The biological replicates were then averaged and fold induction was determined in a $2^{-\Delta\Delta Ct}$ -based fold-change calculation.

Determination of ALT in plasma

Blood was obtained by cardiac puncture in the post-mortem left atrium with a heparinized syringe (5% heparin solution in saline), and collected in 1.5 mL tubes with 10 µL of heparin. The tubes were centrifuged at 300x *g* for 30 min at 4 ºC and the plasma (upper phase) was collected in new tubes, and then frozen at -80 ºC. Plasma alanine transaminase (ALT) activity was determined with a specific kit from BioSystems (Wako Chemicals GmbH, Neuss, Germany).

Histopathology assessment

Hematoxylin-Eosin (H&E) paraffin-embedded liver biopsy sections from studied mice were evaluated by an experienced liver pathologist (Echevarne Laboratories, Valencia, Spain) blinded to the features of animal groups. Samples of livers were fixed overnight in 4% PFA, embedded in paraffin and sectioned 3 µm thick. Slides were dewaxed and re-hydrated through a series of graded ethanol until water and were stained with H&E. A semi-quantitative analysis has been used to estimate the extension of parenchymal necrosis. At least five fields of each sample (4-8 animals per condition) were examined at low power fields, 100X magnifications. Scores used were 0=none, 1=mild lesion (0-20% of necrosis), 2= moderate lesion (20-40% of necrosis), 3=severe lesion (more than 40% of necrosis) as described in [28]. Photographs were taken using an Olympus DP73 digital camera.

Total NAD and NADH content determination

For determination of total NAD and NADH content, the NAD/NADH Assay Kit (Colorimetric) was used (ab65348, Abcam). Briefly, frozen liver pieces of minimum 20 mg were homogenized in the corresponding NAD/NADH extraction buffer at a ratio of 400 µL/20 mg in a glass-potter. Deprotienization of the samples was performed with 10 kDa spin columns. Total NAD and NADH samples were loaded in duplicates in a 96-well flat-bottom plate and the assay was performed according to the manufacturer's instructions. Absorbance was measured at 450 nm in a Spark TECAN microplate reader until the OD stabilized (Tecan, Männedorf, Switzerland).

AMP and ATP determination

The determination of AMP content was performed with the AMP Assay kit (Colorimetric) (ab273275, Abcam). Frozen liver tissue (minimum 10 mg) was homogenized in AMP assay buffer at a ratio of 100 µL/10 mg in a glass-potter. Deprotienization of the samples was performed with 10 kDa spin columns and the filtrate was loaded onto a 96-well flat-bottom plate in duplicate. The assay was

performed according to the manufacturer's instructions and absorbance was measured at 560 nm in a Spark TECAN microplate reader.

Detection of total ATP was analyzed with the ATP colorimetric/fluorometric assay (MAK190, Sigma). 10 mg of liver tissues were homogenized in 100 μL of ATP assay buffer in a glass-potter. The samples were diluted with ATP assay buffer and loaded to a black 96-well flat-bottom plate, in triplicate. The assay was performed according to the manufacturer's instructions. Fluorometry was measured with a Spark TECAN microplate reader, **λ**Ex=535 nm, **λ**Em=587 nm.

Blue Native (BN)-PAGE Analysis

Isolated mitochondria were centrifuged at 7000x *g* for 10 min at 4 °C. The mitochondria pellet was resuspended in an appropriate volume of resuspension buffer (1 M 6-aminohexanoic acid, 50 mM Bis-Tris (HCl), pH 7.0) to a final mitochondrial concentration of 10 μg/μL. 100 μg of mitochondria were incubated 5 min on ice with 10% digitonin (10% (w/v) digitonin in 50 mM NaCl, 50 mM imidazole, 5 mM 6-aminohexanoic acid, 4 mM PMSF), and then centrifuged at maximum speed for 30 min at 4 °C. The supernatant was collected in a new tube and blue sample buffer (5% (w/v) blue G dye, 1 M 6-aminohexanoic acid) was added (20% of the final volume). Samples were loaded in the XCellSureLock Mini-Cell Electrophoresis System (Thermo Fisher Scientific); dark blue cathode buffer (20% (w/v) comassie brilliant blue G250 in anode buffer) was loaded on the inside and anode buffer (NativePAGE Running Buffer 20x, Thermo Fisher Scientific) in the outside. The gel was run 30 min at 150 V at 4 ºC, then the dark blue cathode buffer was replaced with light blue cathode buffer (1/10 dilution dark blue cathode buffer : anode buffer), and run for an additional 60 to 150 min at 250V at 4 ºC. At the end of the run, the gel was stained with blueSafe (NZYTech) and scanned or transferred to a PVDF membrane for immunoblotting assays.

ROS production evaluation

For the evaluation of ROS production, H_2O_2 production was recorded at the same time as oxygen consumption. The system was calibrated before use at 37 ºC with Mir05 medium. The system was also calibrated with titrations of 0.1 μM of H_2O_2 in the presence of superoxide dismutase, SOD (5 U/mL), horseradish peroxidase, HRP (1 U/mL) and amplex ultra red (10 μM), to generate a standard curve. Amplex red, together with H_2O_2, serves as a substrate for HRP to generate a fluorescent molecule, which is recorded by the system. The fluorescence signal is proportional to the H_2O_2 produced. Once the system was calibrated, 50 μg of mitochondria were added, and then different substrates were added sequentially: 10 mM succinate, 5 mM ADP and 5 mM pyruvate. Finally, 0.5 μM rotenone was added as Complex I inhibitor and 2.5 μM antimycin A as Complex III inhibitor. The fluorescent signal (i.e. H_2O_2 production), as well as oxygen concentrations and oxygen flux data were processed by DatLab 7.4 software. H_2O_2 flow (amoL/s·mg) and Specific Flux (pmoL/s·mg) data were obtained. The ratio of H_2O_2 flow to O_2 flow was also calculated.

Supplementary Tables

Supplementary Table s1. Antibodies used in Western Blot

Primary Antibody	Host specie	Dilution	Reference	Secondary Antibody	Host specie	Dilution	Reference
OXPHOS	mouse	1:250	ab110413 Abcam	Anti-mouse IgG HRP	Goat	1:10000	10004302 Cayman
CytC	mouse	1:1000	45-6100 ThermoFisher	Anti-mouse IgG HRP	Goat	1:10000	10004302 Cayman
NDFUA5	rabbit	1:1000	16640-1-AP Proteintech	Anti-rabbit IgG HRP	Goat	1:10000	10004301 Cayman
MFN1	rabbit	1:1000	13798-1-AP Proteintech	Anti-rabbit IgG HRP	Goat	1:10000	10004301 Cayman
MFN2	mouse	1:1000	ab56889 Abcam	Anti-mouse IgG HRP	Goat	1:10000	10004302 Cayman
DRP1	rabbit	1:500	ab184247 Abcam	Anti-rabbit IgG HRP	Goat	1:10000	10004301 Cayman
OPA1	rabbit	1:1000	ab15457 Abcam	Anti-rabbit IgG HRP	Goat	1:10000	10004301 Cayman
FIS1	rabbit	1:1000	10956-1-AP Proteintech	Anti-rabbit IgG HRP	Goat	1:10000	10004301 Cayman
YME1L1	rabbit	1:1000	11510-1-AP Proteintech	Anti-rabbit IgG HRP	Goat	1:10000	10004301 Cayman
OMA1	mouse	1:1000	sc-515788 Santa Cruz	Anti-mouse IgG HRP	Goat	1:5000	10004302 Cayman

Supplementary Table s2. Primer sequences used for qPCR.

Gene	Primer pair	Technology
ApoB	FP: 5' CGTGGGCTCCAGCATTCTA 3'<br>RP: 5' TCACCAGTCATTTCTGCCTTTG 3'	SYBRGreen
CytB	FP: 5' GCTTTCCACTTCATCTTACCATTTA 3'<br>RP: 5' TGTTGGGTTGTTTGATCCTG 3'	SYBRGreen
Hprt	FP: 5' TCCTCCTCAGACCGCTTTT 3'<br>RP: 5' CCTGGTTCATCATCGCTAATC 3'	TaqMan<br>(probe #95, UPL Roche)
Tfam	FP: 5' TGAGGCTTGGAAAAATCTGTCT 3'<br>RP: 3' TCGTCCAACTTCAGCCATC 3'	TaqMan<br>(probe #103, UPL Roche)
Pgc1α	FP: 5' AATTTTTCAAGTCTAACTATGCAGACC 3'<br>RP: 5' CAAAATCCAGAGAGTCATACTTGC 3'	TaqMan<br>(probe #31, UPL Roche)

Supplementary Table s3. qPCR programs

Fast SYBRGreen

**Temperature**	**Time**	
95ºC	20s	
95ºC	1s	x 40 cycles
60ºC	20s	
95ºC	1s	Melt Curve Stage
60ºC	20s	
95ºC	1s	

TaqMan Reagents

**Temperature**	**Time**	
95ºC	10min	
95ºC	15s	x 45 cycles
60ºC	60s	
72ºC	1s	
40ºC	30s	

Supplementary Figures

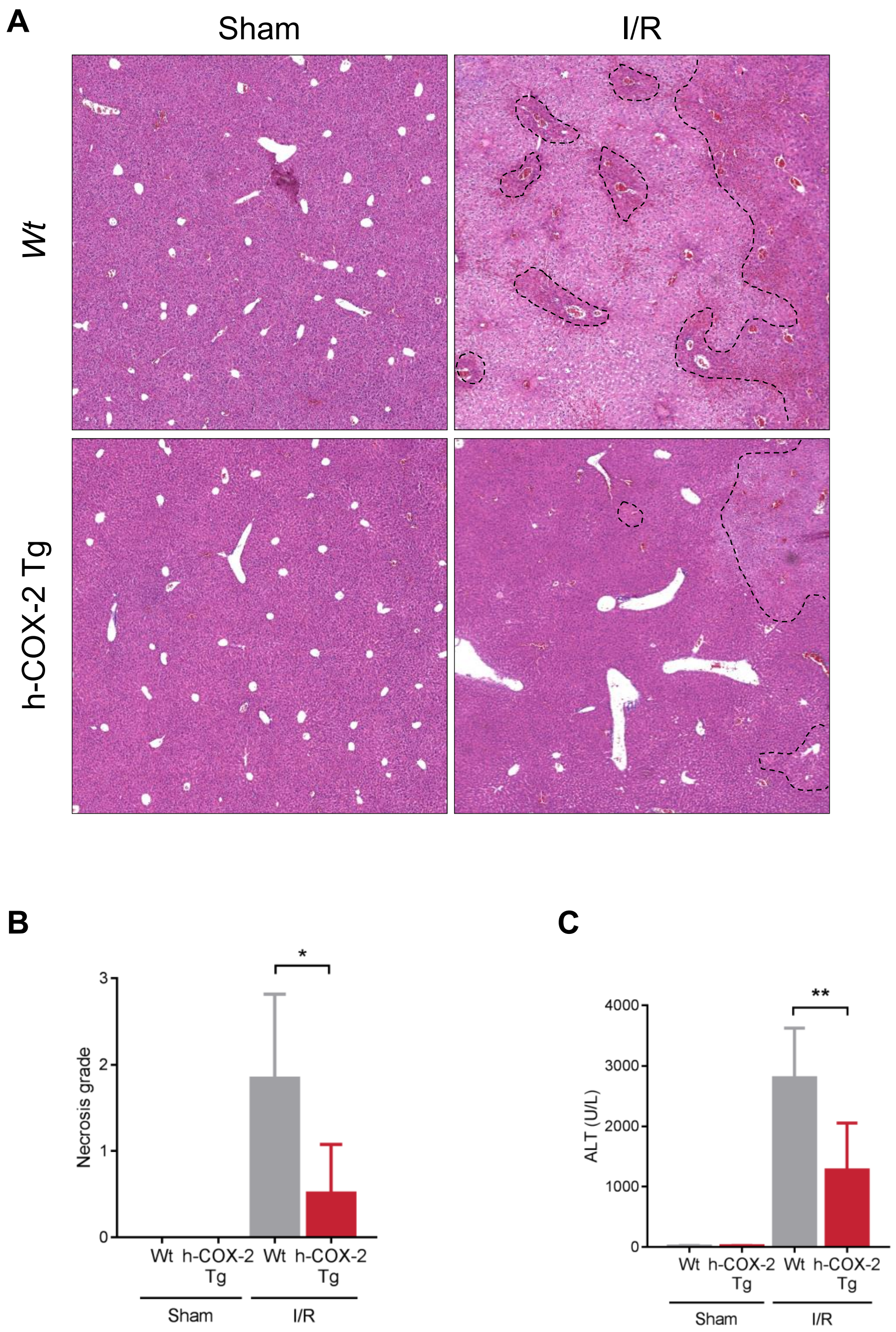

Supplementary Figure s1. Validation of tissue damage after Sham and I/R surgery, comparing Wt and h-COX-2 Tg livers. **(A)** Representative images of H&E staining. **(B)** Necrosis grade. **(C)** Alanine transaminase analysis in plasma. Bars are means ± SD of 4-9 independent measures.

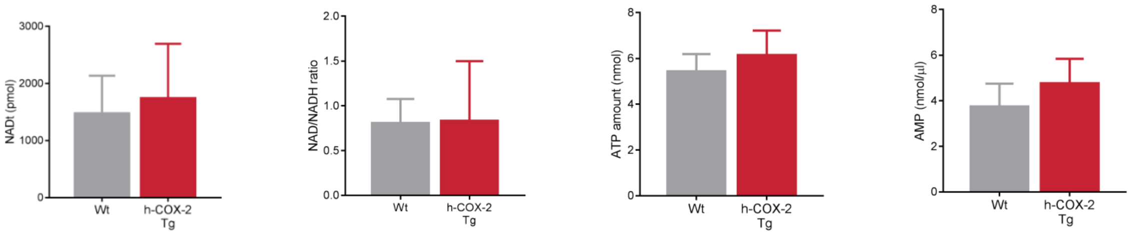

Supplementary Figure s2. Measurement of cofactors in Wt and h-COX-2 Tg livers after I/R surgery. Total NAD (in pmoL), NAD/NADH ratio, ATP (in nmoL) and AMP (in nmoL/μL) levels from liver homogenates were determined following the protocols described in Material and Methods. Bars are means ± SD of 7 independent measured.

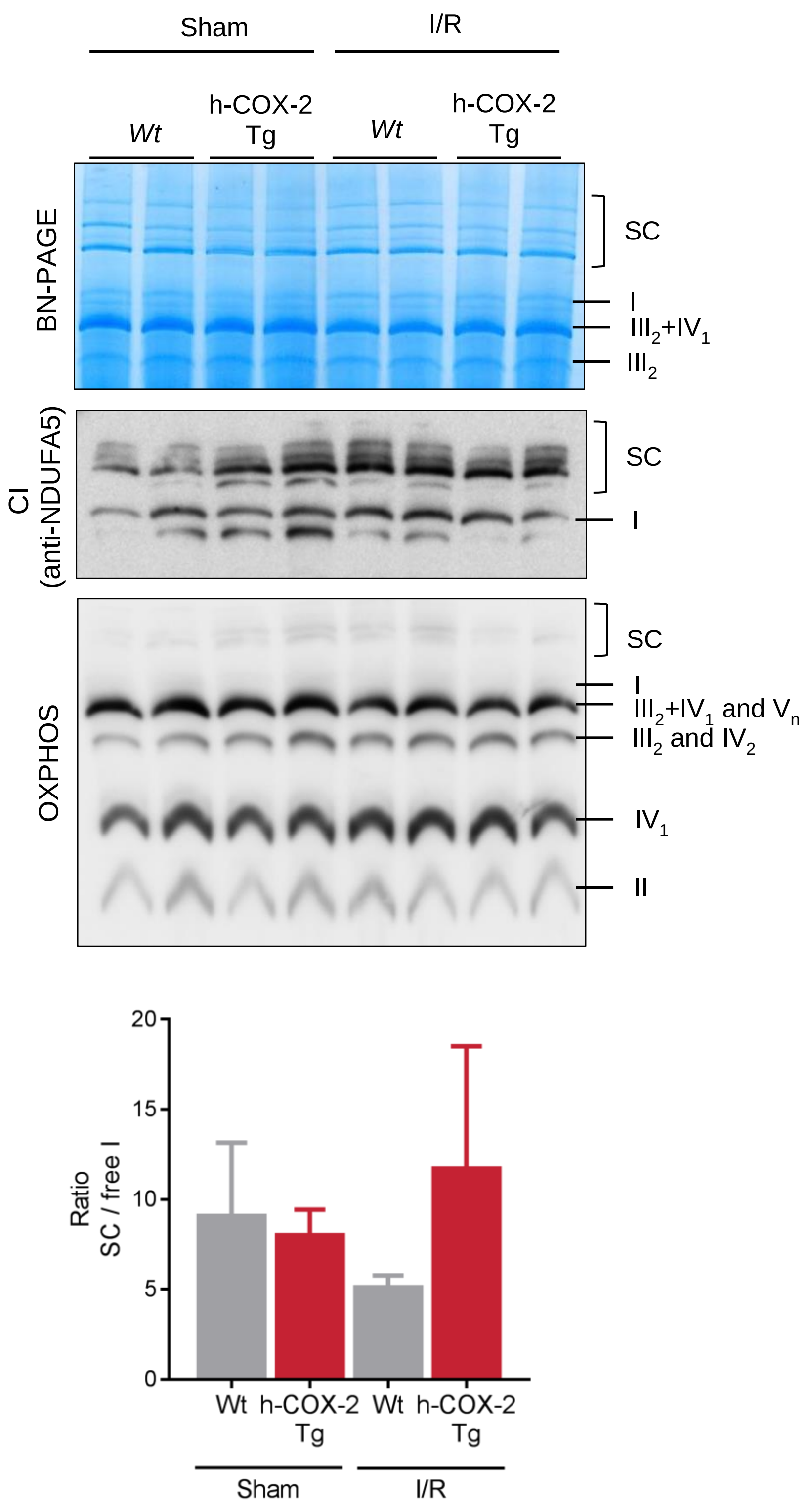

Supplementary Figure s3. Analysis of ETC supercomplexes (SC) in liver-derived mitochondria from Wt and h-COX-2 Tg mice (N=4-9) after Sham or I/R surgery. BN-PAGE and immunoblot representation for CI and total OXPHOS. Bars are means ± SD of 4-9 independent measures.

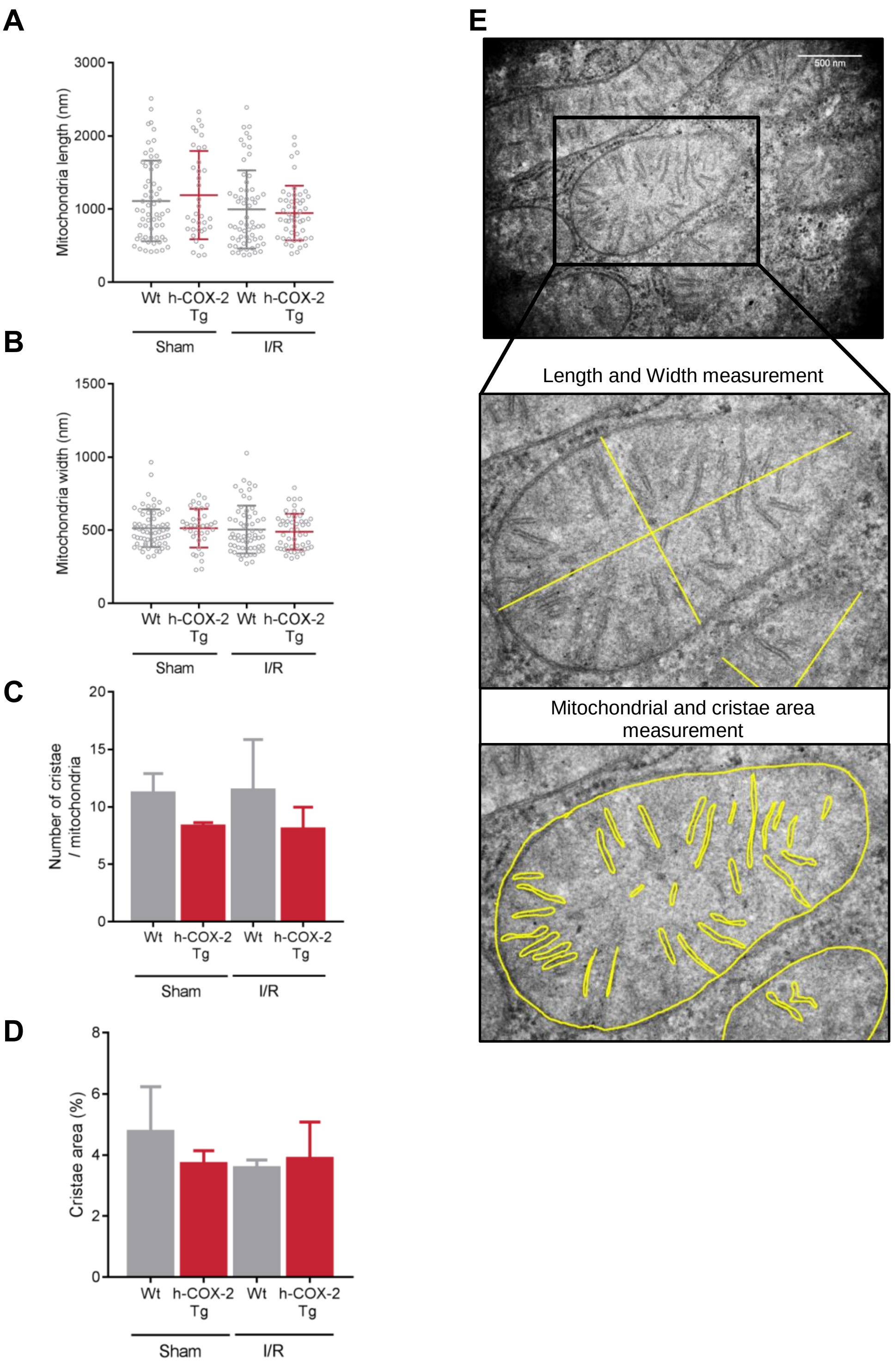

Supplementary Figure s4. Study of mitochondrial cristae in liver tissue after Sham or I/R surgery in livers from Wt and h-COX-2 Tg mice. **(A)** Quantification of mitochondrial length and **(B)** width in nm. Each dot represents 1 mitochondrion. **(C)** Quantification of the number of cristae per mitochondria and **(D)** occupied cristae area in relation to mitochondrial area. **(E)** Representative Transmission Electron Microscopy image showing how mitochondrial measurements were performed (length, width, mitochondrial area and cristae area). Bars are means ± SD of 5 images/animal, from 3-5 animals/condition.

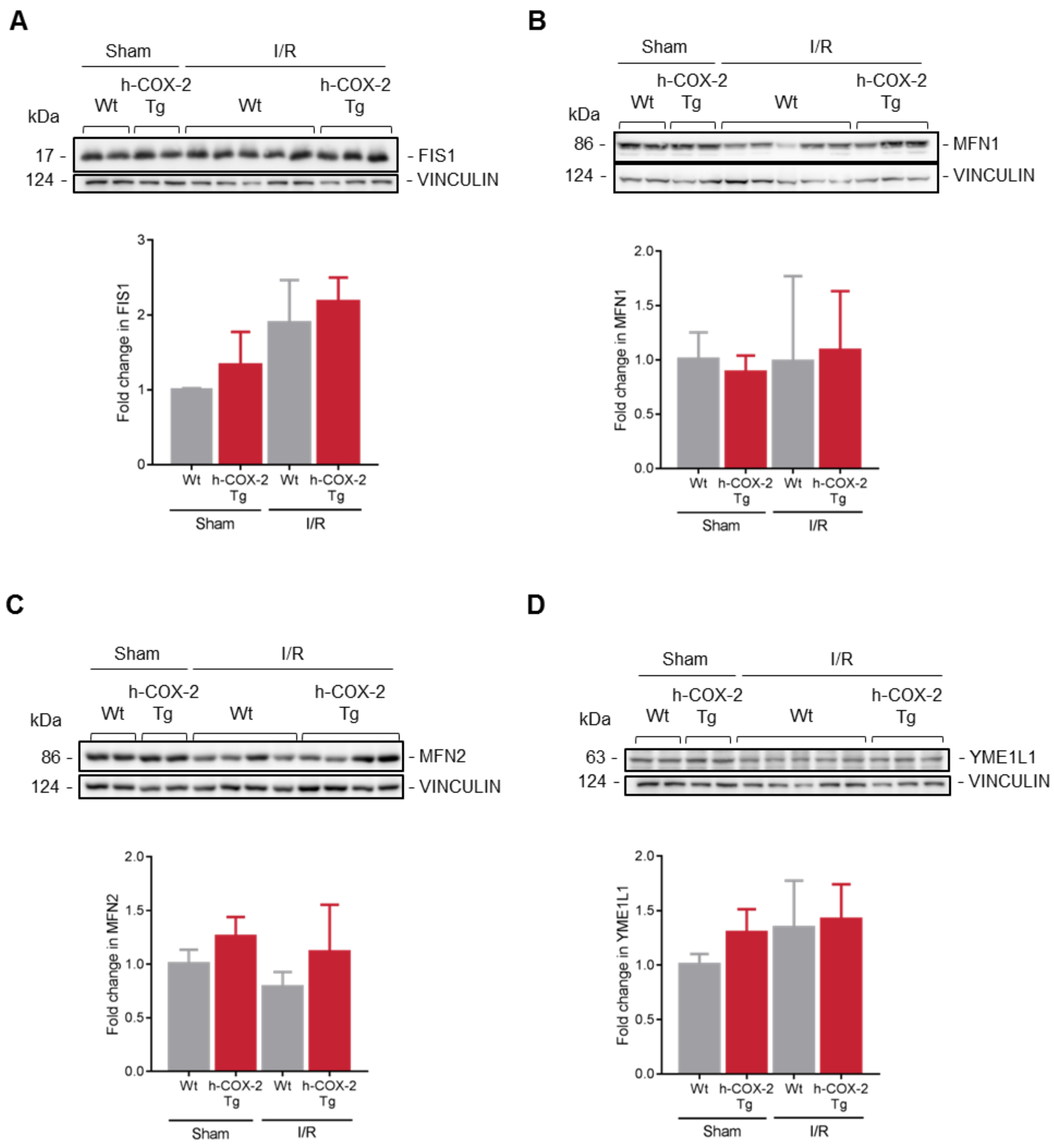

Supplementary Figure s5. Evaluation of fusion/fission-related proteins in Wt and h-COX-2 Tg liver homogenates after Sham or I/R surgery. Representative western blot and densitometry analysis of FIS1 **(A)**, MFN1 **(B)**, MFN2 **(C)** and YME1L1 **(D)**. Bars are means ± SD of 3-9 independent measured.

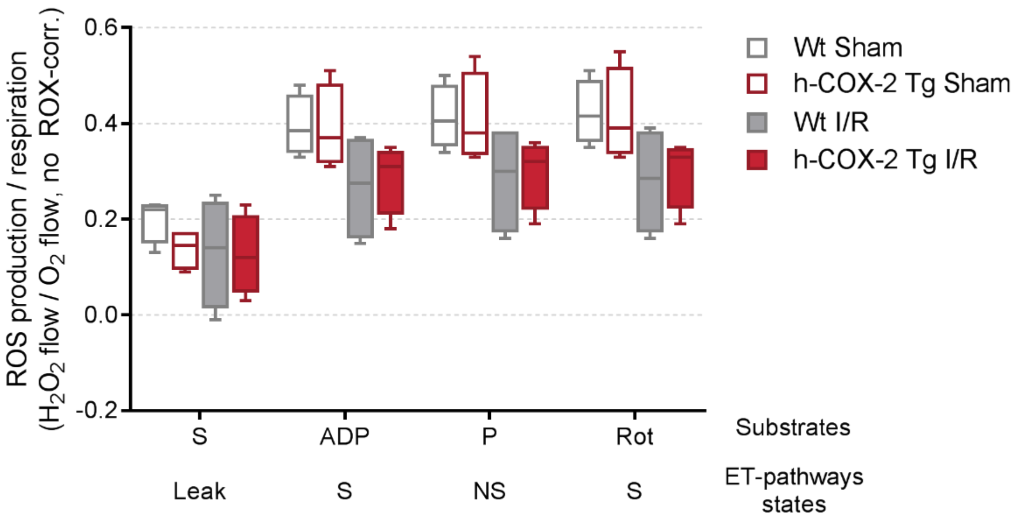

Supplementary Figure s6. Combined determination of oxygen consumption and H_2O_2 flux by O2k-Fluorometry in in mitochondria after Sham and I/R surgery, using succinate as initial substrate. H_2O_2 flow/O_2 flow ratio was calculated (flows are calculated in amoL/s·mg of protein). The fluorescence signals were calibrated using the H_2O_2 titrations at the corresponding state. Bars are means ± SD of 4 independent measures.
